# Evaluation of Direct Collocation Optimal Control Problem Formulations for Solving the Muscle Redundancy Problem

**DOI:** 10.1007/s10439-016-1591-9

**Published:** 2016-03-21

**Authors:** Friedl De Groote, Allison L. Kinney, Anil V. Rao, Benjamin J. Fregly

**Affiliations:** 1 Department of Kinesiology, KU Leuven, Tervuursevest 101 bus 1501, 3001 Leuven, Belgium; 2 Department of Mechanical and Aerospace Engineering, University of Dayton, Dayton, OH USA; 3 Department of Mechanical & Aerospace Engineering, University of Florida, Gainesville, FL USA

**Keywords:** Muscle force estimation, Direct collocation, Optimization, Muscle dynamics, Biomechanics

## Abstract

Estimation of muscle forces during motion involves solving an indeterminate problem (more unknown muscle forces than joint moment constraints), frequently *via* optimization methods. When the dynamics of muscle activation and contraction are modeled for consistency with muscle physiology, the resulting optimization problem is dynamic and challenging to solve. This study sought to identify a robust and computationally efficient formulation for solving these dynamic optimization problems using direct collocation optimal control methods. Four problem formulations were investigated for walking based on both a two and three dimensional model. Formulations differed in the use of either an explicit or implicit representation of contraction dynamics with either muscle length or tendon force as a state variable. The implicit representations introduced additional controls defined as the time derivatives of the states, allowing the nonlinear equations describing contraction dynamics to be imposed as algebraic path constraints, simplifying their evaluation. Problem formulation affected computational speed and robustness to the initial guess. The formulation that used explicit contraction dynamics with muscle length as a state failed to converge in most cases. In contrast, the two formulations that used implicit contraction dynamics converged to an optimal solution in all cases for all initial guesses, with tendon force as a state generally being the fastest. Future work should focus on comparing the present approach to other approaches for computing muscle forces. The present approach lacks some of the major limitations of established methods such as static optimization and computed muscle control while remaining computationally efficient.

**Electronic Supplementary Material:**

The online version of this article (doi:10.1007/s10439-016-1591-9 contains supplementary material, which is available to authorized users.

## Introduction

Knowledge of muscle forces during healthy and impaired movement could facilitate the development of improved treatments for disorders affecting walking ability or improved training programs to increase athlete performance. As a result, significant research effort has been dedicated to estimating muscle forces during normal (e.g.,^1^,^4^,^20^) and impaired (recently e.g.,^13^,^26^,^31^,^34^) movement. Since muscle forces are not directly measurable, these studies have been based on computational models. However, there are many more muscles than degrees of freedom in the human skeleton, and thus the muscle forces underlying a given motion cannot be uniquely calculated using rigid body dynamics. Consequently, optimization methods have been used to resolve this redundancy by assuming that human movement is produced by optimizing some performance criterion.^24^

The numerical challenges arising from the use of optimization methods have led to a trade-off between computational efficiency and consistency with muscle physiology.^5^ When the dynamics of muscle activation and contraction are modeled for consistency with muscle physiology, the resulting optimization problem is dynamic and challenging to solve due to the non-linearity and stiffness of the equations describing muscle dynamics (i.e., muscle activation and contraction dynamics).^29^ Commonly, the dynamic optimization problem is solved using direct shooting (e.g.,^3^,^4^,^18^–^21^). Direct shooting methods parametrize the controls, in this case muscle excitations, and solve for control parameters that optimize the cost function. The cost function is evaluated using time-marching (time frames are solved sequentially) to simulate the dynamic equations. The main disadvantage of time-marching is the high sensitivity of the states to the controls due to the long time interval over which the time-marching method is applied, often resulting in long computation times. Anderson and Pandy,^4^ for example, reported a CPU time of 10,000 h to solve a dynamic optimization problem for half a cycle of walking. Except for simple problems, convergence of dynamic optimization problems is difficult to obtain and the solver is interrupted once an acceptable solution is found (e.g.,^4^,^18^). In addition, simple control parameterizations are often used (e.g.,^20^) that might not accurately describe the optimal solution. Note that in these examples, dynamic optimization was combined with a forward dynamics analysis of skeletal motion. However, others have combined dynamic optimization with an inverse dynamics analysis of skeletal motion (e.g.,^15^). We will use the phrases “musculoskeletal dynamic optimization” and “muscle dynamic optimization” to distinguish between dynamic optimization approaches that account for muscle dynamics in combination with either a forward (musculoskeletal dynamic optimization) or inverse (muscle dynamic optimization) dynamics simulation to account for skeletal dynamics. Due to the use of an inverse dynamics approach, muscle dynamic optimization is only applicable if the motion is prescribed, which is the case considered in this manuscript, whereas musculoskeletal dynamic optimization can be used for both tracking and predicting motion.

Due to the numerical challenges involved in solving dynamic optimization problems, many studies use simple optimization approaches (e.g.,^6^) that neglect muscle activation and contraction dynamics. Neglecting activation and contraction dynamics eliminates coupling between time instants, making the resulting optimization problem static. Static optimization approaches result in a series of small optimization problems, with one problem solved at each time instant. When the sum of squared muscle activations is used as the performance criterion, these optimization problems are quadratic and can be solved very efficiently. These approaches are robust and fast and allow a large community of researchers to estimate muscle forces with the downside of reduced consistency with muscle physiology.^8^

Whether or not reduced consistency with muscle physiology is important is still controversial. Only a few modeling studies have investigated the influence of muscle activation and contraction dynamics on movement ability and performance. Some have argued that modeling muscle activation and contraction dynamics has only a small effect on the computed muscle forces during walking^5^ and even running.^14^ Others, however, have demonstrated that dynamic muscle behavior has a large influence on predicted muscle forces during wheelchair propulsion^17^ and on maximal sprinting performance.^16^ Hence, some research questions might be addressed best by modeling muscle activation and contraction dynamics. A robust and efficient method to solve the dynamic muscle redundancy problem would therefore greatly benefit researchers seeking to understand normal and impaired movement better through assessment of individual muscle function.

Direct collocation is a recent promising methodological improvement over direct shooting to increase the computational efficiency of dynamic optimization approaches.^1^,^2^,^7^,^8^,^29^ In contrast to time-marching, direct collocation simulates the dynamic equations by solving all time frames simultaneously. Both the controls and the states are parameterized and the discretized state equations are solved while optimizing the performance criterion, resulting in a non-linear programming problem (NLP) with a large number of optimization variables as compared to direct shooting methods. The sparsity of these problems, however, makes them tractable, and therefore collocation methods are often more efficient computationally than are shooting methods. However, due to the stiffness of the dynamic equations, solving the NLP arising from a dynamic optimization problem is challenging, and only a few studies have explored this approach. De Groote *et al.* 7 presented a sequential approach to solve the muscle dynamic optimization problem. Their approach approximates non-smooth non-linear dynamic equations by a smooth linear discretization that is updated every iteration. Though computationally efficient, convergence of this approach to a local optimum of the original dynamic optimization problem could not be guaranteed. Van den Bogert *et al.* applied direct collocation to a non-linear musculoskeletal dynamic optimization problem for walking. They obtained fast convergence but did not verify the optimality of their numerical solution,^1^,^2^,^29^ which would provide confidence that direct collocation is an appropriate method for solving the dynamic optimization problems. In both cases, convergence relied heavily on the availability of a good initial guess, making existing direct collocation formulations less suitable for use by non-experts.^29^

This study sought to identify a formulation for solving the muscle dynamic optimization problem using direct collocation optimal control methods that is computationally efficient and robust to the initial guess. Since numerical optimization is sensitive to problem formulation, four optimal control problem formulations were investigated. Each formulation optimized the same performance criterion, modeled activation dynamics, and used either an explicit or implicit representation of contraction dynamics with either normalized muscle fiber length or normalized tendon force as a state variable. The implicit representations introduced additional controls defined as the time derivatives of the states, resulting in very simple dynamic equations and allowing the nonlinear equations describing muscle contraction dynamics to be imposed as algebraic path constraints, simplifying their evaluation. The different problem formulations were evaluated by estimating muscle forces during normal walking using both a simple and a complex musculoskeletal model. The optimality of the solutions obtained was confirmed using two different approaches following the suggestion of Hicks and al.^12^ to verify software used for musculoskeletal modeling and simulation. The robustness and efficiency of the proposed implicit formulations might enable the use of muscle dynamic optimization by non-experts seeking to investigate the effect of muscle dynamics on the efficiency and performance of human movement.

## Materials and Methods

### Musculoskeletal Model

To perform the proposed study, we started with a simple and a complex musculoskeletal model taken from the Models folder installed with OpenSim 3.2.^9^ The simple model (gait10dof18musc) contained three degrees of freedom (hip, knee, and ankle angle in the sagittal plane) and nine muscles per leg, while the complex model (gait2392) contained five degrees of freedom (three at the hip and one at the knee and ankle) and 43 muscles per leg.

**Figure 1:**
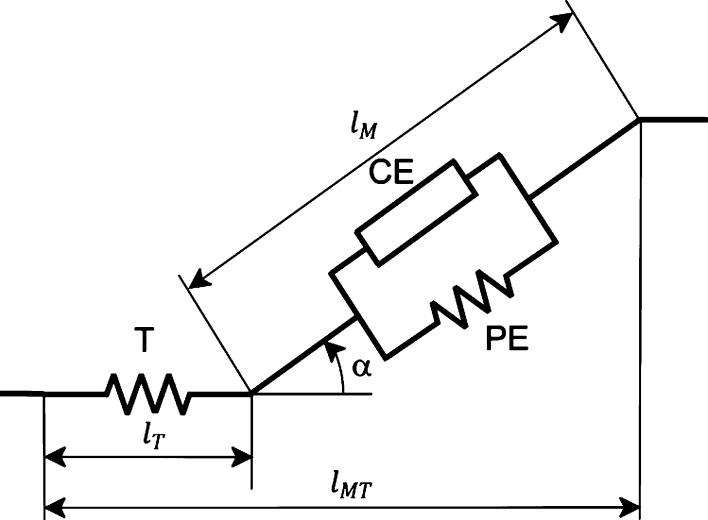
Schematic representation of the Hill muscle model.^35^ A first-order lumped parameter model accounting for the interaction of the force–length–velocity properties of muscle and the elastic properties of tendon. The MT-actuator comprises a tendon, T, in series with a muscle. The muscle consists of a contractile element, CE, parallel to a passive element, PE. The tendon is modeled as a nonlinear spring. *l*_M_ is muscle fiber length, *l*_T_ is tendon length, and *l*_MT_ is muscle-tendon length. The pennation angle *α* is the angle between the orientation of the muscle fibers and the tendon.

**Figure 2:**
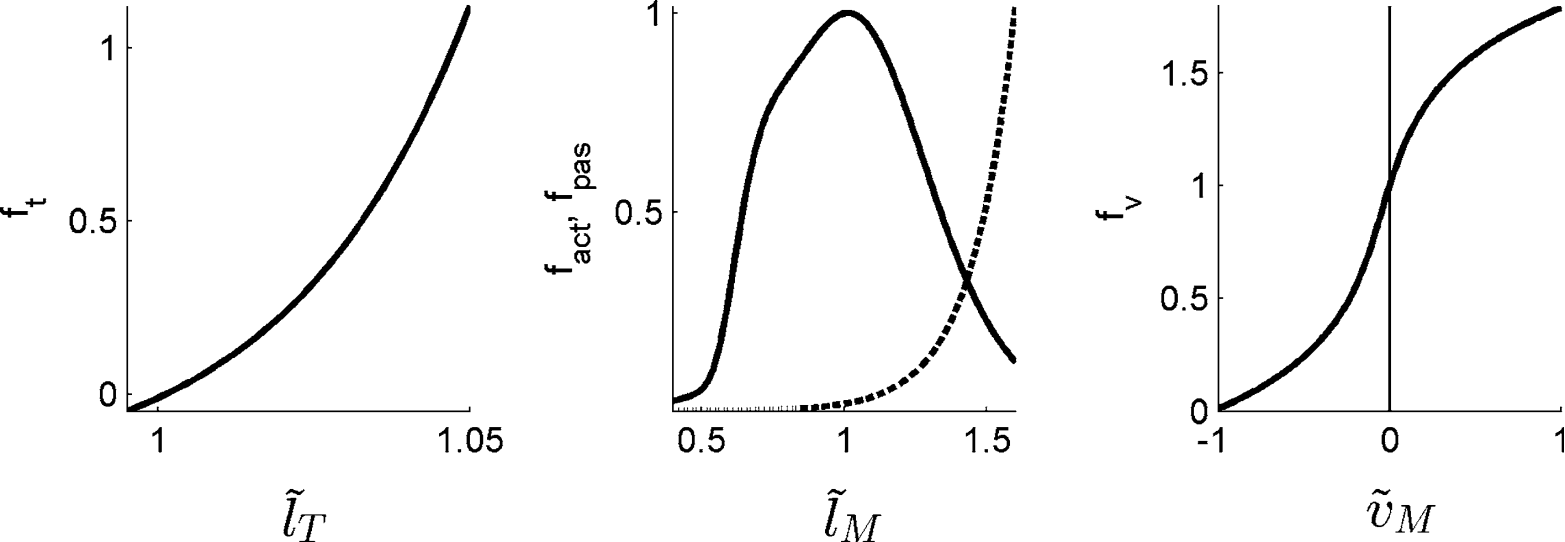
Characteristics describing normalized muscle-tendon properties. Tendon force-length relationship $f_{t} (\tilde{l}_{\text{T}} )$ with $\tilde{l}_{\text{T}}$ normalized tendon length (left), active (solid line) and passive (dashed line) muscle force-length relationships $f_{\text{act}} (\tilde{l}_{\text{M}} )$ and $f_{\text{pas}} (\tilde{l}_{\text{M}} )$ with $\tilde{l}_{\text{M}}$ normalized muscle length (middle), and muscle force-velocity relationship $f_{\text{v}} (\tilde{v}_{\text{M}} )$ with $\tilde{v}_{\text{M}}$ normalized muscle velocity (right). Negative tendon forces are non-physiological but will never occur when muscle and tendon force are equilibrated (Eq. 7), since muscle force cannot drop below zero (Eq. 4). This modification of *f*_*t*_ makes the solution of Eqs. (3)–(7) better conditioned when the muscle-tendon actuator is slack (zero tendon force corresponds to normalized tendon length of 1 rather than a whole range of tendon lengths).

Each muscle in the model was represented as a Hill-type muscle-tendon unit^35^ (Fig. 1). Muscle dynamics was described by two nonlinear, first order differential equations—activation and contraction dynamics—that relate the control—muscle excitation—to the states—muscle activation and either normalized fiber length or normalized tendon force. Activation dynamics was modeled based on Winters,^27^,^33^ using a tanh function to smoothly transition between activation and deactivation:

1 $$f = 0.5 \tanh (b(e - a)),$$

2 $$\frac{da}{dt} = \left[ {\frac{1}{{\tau_{a} \left( {0.5 + 1.5a} \right)}}\left( {f + 0.5} \right) + \frac{0.5 + 1.5a}{{\tau_{d} }}\left( { - f + 0.5} \right)} \right]\left( {e - a} \right),$$

where *e* is muscle excitation, *a* is muscle activation, *τ*_*a*_ = 0.015 *s* is the activation time constant, *τ*_*d*_ = 0.060 *s* is the deactivation time constant, and *b* = 0.1 is a parameter determining transition smoothness. Contraction dynamics was described based on Hill’s model^35^ (Fig. 1). The muscle-tendon actuator consisted of a tendon with length *l*_T_ in series with a muscle with fiber length *l*_M_, where the pennation angle *α* defines the angle between the tendon and the muscle fibers. Properties of muscle and tendon were described by dimensionless characteristics (Fig. 2). Five parameters scaled these generic characteristics for a specific muscle: optimal fiber length $l_{\text{M}}^{0}$, maximal muscle fiber velocity $v_{\text{M}}^{\hbox{max} }$, peak isometric muscle force $F_{\text{M}}^{0}$, tendon slack length $l_{\text{T}}^{s}$, and pennation angle at optimal fiber length *α*_0_. Values for these five parameters were taken from the OpenSim models described above. Tendon was modeled by a nonlinear spring:

3 $$F_{\text{T}} = F_{\text{M}}^{0} f_{\text{t}} \left( {\tilde{l}_{\text{T}} } \right),$$

where *F*_T_ is tendon force, $\tilde{l}_{\text{T}} = l_{\text{T}} /l_{\text{T}}^{s}$ is normalized tendon length and *f*_t_ is the tendon force-length characteristic (see online supplement for mathematical expression). Muscle was modeled by a contractile element in parallel with a passive element:

4 $$F_{\text{M}} = F_{\text{M}}^{0} \left[ {af_{\text{act}} \left( {\tilde{l}_{\text{M}} } \right)f_{\text{v}} \left( {\tilde{v}_{\text{M}} } \right) + f_{\text{pas}} \left( {\tilde{l}_{\text{M}} } \right)} \right],$$

where *F*_M_ is muscle force, $\tilde{l}_{\text{M}} = l_{\text{M}} /l_{\text{M}}^{o}$ is normalized fiber length, $\tilde{v}_{\text{M}} = v_{\text{M}} /v_{\text{M}}^{\hbox{max} }$ is normalized fiber velocity, and *f*_act_, *f*_pas_, and *f*_v_ are the active muscle force-length, passive muscle force-length, and muscle force-velocity characteristics, respectively (see online supplement for mathematical expressions). The interaction between muscle and tendon was described by (Fig. 1):

5 $$l_{\text{MT}} = l_{\text{T}} + l_{\text{M}} \cos \alpha ,$$

6 $$l_{\text{M}} \sin \alpha = l_{\text{M}}^{0} \sin \alpha_{0} ,$$

7 $$F_{\text{T}} = F_{\text{M}} \cos \alpha .$$

The five Eqs. (3)–(7) determine the five unknowns *F*_T_, *F*_M_, *l*_T_, *l*_M_, *α*, given the input *a* and the muscle-tendon length *l*_MT_. The dynamic nature of the Hill model results from the fiber velocity dependence of Eq. (4). Note, however, that under the assumption of a rigid tendon and hence constant tendon length $l_{T} = l_{T}^{s}$, muscle fiber length and velocity are completely determined by muscle-tendon length *l*_MT_ and velocity *v*_MT_ (Eqs. 5, 6), which can be computed from skeletal kinematics, thereby allowing algebraic solution of Eqs. (3)–(7).

Given the algebraic relationship between muscle fiber length and tendon force, it is equally valid to choose muscle length or tendon force as the state variable when solving Eqs. (3)–(7).^25^ All characteristics are at least second order continuous and *f*_*t*_ is at least third order continuous (Fig. 2). For numerical reasons, *f*_*t*_ is allowed to be less than zero instead of equal to zero when the tendon is slack. Negative tendon forces are non-physiological but will never occur when muscle and tendon force are equilibrated (Eq. 7), since muscle force cannot drop below zero (Eq. 4). This modification of *f*_*t*_ makes the solution of Eqs. (3)–(7) better conditioned when the muscle-tendon actuator is slack (zero tendon force corresponds to normalized tendon length of 1 rather than a whole range of tendon lengths).

### Experimental Data and Data Processing

**Figure 3:**
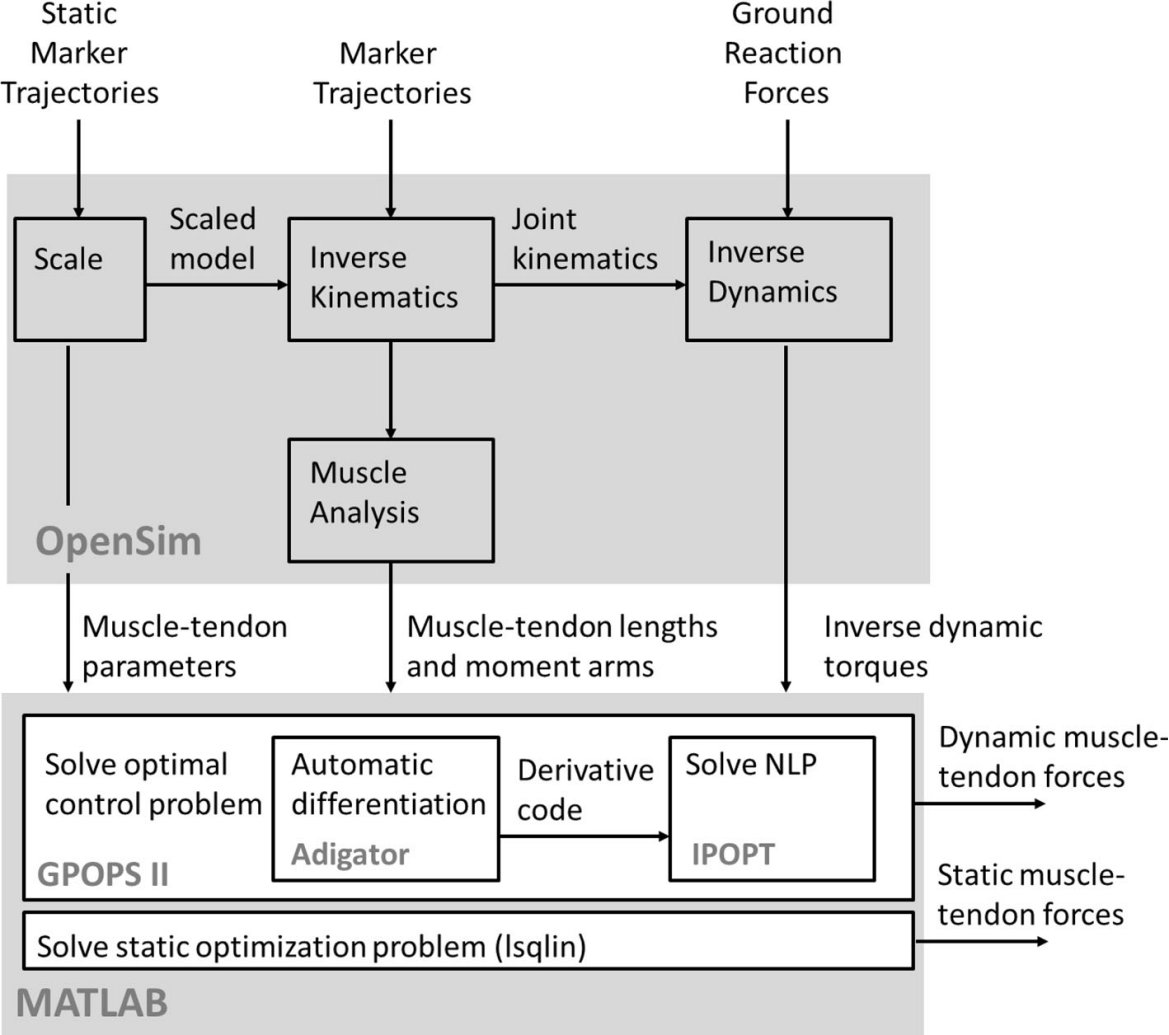
Block diagram illustrating the process and software used to solve the muscle redundancy problem. Setup-files for OpenSim’s Scale and Inverse Kinematics Tools were taken from the Model folder installed with OpenSim 3.2. The Inverse Dynamics Tool was set up to filter the coordinates using a frequency of 6 Hz.

Experimental data for one walking cycle were taken from the Models folder installed with OpenSim 3.2, since the availability of this dataset allows other researchers to compare their methods to the one presented in this paper. Experimental marker trajectories were sampled at 60 Hz. The exact same experimental data were used for the simple and complex model. The muscle force distribution underlying this walking motion was computed for the right limb of both models by combining dynamic optimization with an inverse dynamics analysis of skeletal motion where measured joint kinematics and external (ground reaction) forces were inputs and the joint reaction torques were outputs.^7^,^15^ The inverse dynamics joint torques along with the muscle-tendon lengths and velocities and the muscle moment arms were calculated using the standard workflow in OpenSim 3.2 and used as inputs for the dynamic optimization problems described below (see Fig. 3 for more details). These problems were solved for the controls and states (see below for a formulation-dependent definition) over the motion cycle. The initial and final states, however, are unknown. We found that the initial and final states only influenced the optimal controls and states over a period of about 50 ms at the beginning and end of the time interval over which the dynamic optimization problem was solved. Therefore, problems were solved for a time interval containing five additional data points at the beginning and end of the motion cycle to limit the influence of the unknown initial and final state (the final state influences the optimal control at preceding time instants) on the solution for the motion cycle under consideration and results for these additional data points were not reported.

### Problem Formulations and Solution Method

The goal of each optimization problem was to find muscle excitations bounded between 0 and 1 that produced the specified inverse dynamics joint torques while minimizing the integral of the sum of squared excitations for all muscles over the duration of the motion. The use of a quadratic cost functional was first proposed by Pedotti at al.^24^ and is a measure of muscular effort. Activation and contraction dynamics relate muscle excitations to muscle forces whereas the pre-computed muscle moment arms relate muscle forces to joint torques. For each degree of freedom, an ideal actuator that can produce torque instantaneously was added to the model to guarantee problem feasibility in the presence of modeling and measurement errors. The use of these non-physiological actuators was discouraged by weighting their contribution heavily in the cost function. This approach resulted in the following dynamic optimization problems.

#### Cost Functional

The cost functional consisted of two terms. The first term represented muscular effort modeled by the integral of the sum of squared muscle excitations, whereas the second term penalized the use of the non-physiological ideal torque actuators:

8 $$\mathop \smallint \limits_{{t_{0} }}^{{t_{f} }} \left( {\mathop \sum \limits_{m = 1}^{M} e_{m}^{2} + w\mathop \sum \limits_{k = 1}^{K} e_{Tk}^{2} } \right)dt$$

where *t* is time, *t*_0_ and *t*_f_ are the initial and final time, respectively, *m* = 1,…,*M* indicates the different muscles, *e*_T*k*_ are the inputs for the ideal actuators, *k* = 1,…,*K* indicates the different degrees of freedom, and *w* = 1000 is a weight penalizing the use of the non-physiological ideal actuators. This weight was chosen such that the contribution of the ideal torque actuators is below 1 Nm for walking, which we think is acceptable given measurement and modeling uncertainties.

#### Bounds

Muscle excitations were bounded between 0 and 1 whereas the ideal torque actuators could generate both positive and negative torques:

9 $$0 \le e_{m} \le 1$$

10 $$- 1 \le e_{{{\text{T}}k}} \le 1$$

for *m* = 1,…, *M* and *k* = 1,…, *K*, respectively.

#### Path Constraints

The pre-computed muscle moment arms related the muscle forces to the inverse dynamics joint reaction torques:

11 $$T_{{{\text{ID}}k}} = \mathop \sum \limits_{m = 1}^{M} d_{mk} F_{{{\text{T}}m}} + e_{{{\text{T}}k}} T_{\hbox{max} }$$

for *k* = 1,…,*K*, where *T*_ID*k*_ is the inverse dynamics joint torque, *d*_*mk*_ is the moment arm of muscle *m* with respect to the *k*th degree of freedom, and *T*_max_ = 150 Nm is the maximal torque output of the ideal actuators. *T*_max_ was chosen to have the same order of magnitude as the maximal joint torques exerted during the motion to guarantee feasibility of the dynamic optimization problem.

#### Constraints Imposing Muscle Dynamics

Activation dynamics was imposed using Eqs. (1)–(2). Contraction dynamics was imposed using four different formulations as described below:

1. Using normalized tendon force $\tilde{F}_{\text{T}} = \frac{{F_{\text{T}} }}{{F_{\text{M}}^{0} }}$ as a state:
  12 $$\frac{{d\tilde{F}_{\text{T}} }}{dt} = f_{1} (a,\tilde{F}_{\text{T}} ).$$
2. Using normalized muscle fiber length as a state:
  13 $$\frac{{d\tilde{l}_{\text{M}} }}{dt} = f_{2} (a,\tilde{l}_{\text{M}} ).$$
  This formulation of contraction dynamics was typically used in previous methods (e.g.,^9^,^29^).
3. Using normalized tendon force as a state and introducing *u*_F_, the scaled time derivative of the normalized tendon force, as a new control simplifying the contraction dynamic equations:
  14 $$\frac{{d\tilde{F}_{\text{T}} }}{dt} = s_{\text{F}} u_{\text{F}} ,$$
  where *s*_F_ = 10 is a scaling factor. The scaling factor was chosen such that the controls *u*_F_ had the same order of magnitude as the other controls and the states. The Hill model was then imposed as a path constraint:
  15 $$f_{3} \left( {a,\tilde{F}_{\text{T}} ,u_{\text{F}} } \right) = 0.$$
4. Using normalized muscle fiber length as a state and introducing *u*_v_, the scaled time derivative of the normalized muscle length, as a new control simplifying the contraction dynamic equations:
  16 $$\frac{{d\tilde{l}_{\text{M}} }}{dt} = \frac{{v_{\text{M}}^{\hbox{max} } }}{{l_{\text{M}}^{O} }}u_{\text{v}} .$$
  where $v_{\text{M}}^{ \hbox{max} } /l_{\text{M}}^{0}$ is a scaling factor that converts *u*_*v*_, normalized muscle fiber velocity $\tilde{v}_{\text{M}} = \frac{{v_{\text{M}} }}{{v_{\text{M}}^{\hbox{max} } }}$, into the first time derivative of normalized muscle fiber length. Note that normalized muscle fiber velocity is not the first time derivative of normalized muscle length unless normalized time is being used. The Hill model was then imposed as a path constraint:
  17 $$f_{4} \left( {a,\tilde{l}_{\text{M}} ,u_{\text{v}} } \right) = 0.$$

All functions *f*_*i*_, *i* = 1,…,4, were derived from the Hill model described by Eqs. (3)–(7) (see online supplement for full-form expressions). In formulation 2 and 4, which use normalized muscle fiber length as a state, *F*_T_ was computed from $\tilde{l}_{\text{M}}$ based on Eqs. (5), (6), and (3) to evaluate joint torques (Eq. 11). Evaluating *f*_1_ and *f*_2_ required dividing by muscle activation. Muscle activation was bounded between 0.01 and 1 for all formulations to allow comparison of the solutions obtained with the different formulations. The optimal controls and cost function are only expected to be identical if the optimization problems are equivalent, which would not be the case if the states were bounded differently. Normalized muscle forces were bounded between 0 and 3, normalized muscle fiber lengths were bounded between 0.4 and 1.6, controls *u*_F_ were bounded between −50 and 50, and controls *u*_*v*_ were bounded between −1 and 1. At the optimal solution, only the bounds on muscle excitations and muscle activations were active. The feasible set of formulations 3 and 4 differs from the feasible set of formulations 1 and 2 due to the bounds on the additional controls. However, unless these bounds are active at the optimal solution, all formulations have the same globally optimal muscle excitations. Initial and final states were constrained to be within the bounds specified for the states but were not prescribed.

The four muscle dynamic optimization problems were solved numerically through direct collocation using GPOPS-II optimal control software.^22^ GPOPS-II is a MATLAB program that transcribes the dynamic optimization problem to a NLP using a Legendre-Guass-Radau (LGR) quadrature collocation method. All problems were solved on a mesh with 100 equally spaced intervals using third order LGR collocation. Analysis of the mesh accuracy (see below) showed that a further increase in the number of mesh intervals had only a small influence on the optimal solution. The interior point solver IPOPT^30^ was used to solve the resulting large-scale NLPs using second derivative information with a NLP relative error tolerance of 1*e*−6 and a maximum of 2000 iterations. The open-source automatic differentiation software ADiGator^23^ was used to generate derivative source code for use by IPOPT. Automatic differentiation generates analytic derivatives of general functions defined by computer code by applying differentiation rules (e.g., product, quotient, and chain rules) on the elementary function operations that underlie the code.^23^ All computations were performed on an Intel Core i7-4600U 2.1 GHz processor with 16 GB RAM. This computation process is illustrated by the block diagram in Fig. 3.

### Analysis of Results

The four problem formulations were evaluated by estimating muscle forces over one walking cycle using both the simple and complex musculoskeletal model. Convergence, optimal cost function values, mesh accuracy, and CPU times for the different formulations were compared. Mesh accuracy was studied by calculating the root mean square (RMS) difference between the excitations calculated using 100 and 200 mesh intervals, respectively. Solution robustness against changes in the initial guess for the controls and the states was also investigated. Robustness was defined as the RMS difference between excitations calculated using a hot start and an arbitrary initial guess. The hot start was obtained from muscle activations calculated using a previously proposed approach that accounted for activation dynamics but not contraction dynamics.^8^ These activations were used as the initial guess for both the muscle excitations and activations. Dynamically consistent muscle fiber lengths and muscle forces as well as muscle velocities and time derivatives of muscle forces were computed based on contraction dynamics using the initial guess for the activations as an input. These quantities were used as the initial guess for the other controls and states. The arbitrary initial guess consisted of constant values for all controls and states (initial guess of 0.2 for excitations, activations, and normalized tendon force; initial guess of 1 for normalized fiber lengths, initial guess of 0 for all other controls and states). In addition, the effect of bounding muscle activations between 0 and 1 instead of between 0.01 and 1 on the CPU time and mesh accuracy for the third and fourth formulations, which do not require division by muscle activation, was investigated.

Optimality of the results was verified two ways. First, a post-optimality analysis as described in detail by Graham and Rao^11^ was performed to investigate the proximity of the numerical solution to the true optimal solution of the dynamic optimization problem. To this end, the first-order optimality condition that the costate is the sensitivity of the cost with respect to the state along the optimal solution was verified based on the equivalence between the NLP and calculus of variations optimality conditions for LRG collocation methods. A discrete approximation of the costate of the dynamic optimization problem was obtained by a linear transformation of the Lagrange multipliers of the NLP arising from LGR collocation,^10^ and this computation is automatically performed by GPOPS-II when solving an optimization problem. To perform this post-optimality analysis, the dynamic optimization problem was resolved over the walking cycle imposing the previously obtained solution at the beginning of the walking cycle as the initial state. The sensitivity of the cost with respect to the initial state was approximated by resolving the dynamic optimization problem using a perturbed initial state and computing the ratio of the change in cost to the change in initial state. By comparing the costate approximations at the initial time with the sensitivity of the cost to changes of 0.001 in the initial activation of each muscle, we evaluated the optimality of the obtained solutions. For this analysis, GPOPS-II’s mesh refinement algorithm was used. Since contraction dynamics was imposed as a path constraint in formulations 3 and 4 and GPOPS-II’s mesh refinement algorithm does not account for path constraints, the mesh was refined based on activation dynamics accuracy only. This post-optimality analysis was performed for formulations 3 and 4 only, since formulations 1 and 2 did not always converge, and only for the simple model, since problem formulation and solution methods are not model-specific and computation times were much lower for the simple model.

Second, a less formal verification was performed by using the equivalence between static and dynamic optimization in the limit of zero activation and deactivation time constants and infinite tendon stiffness. Since the static optimization problem is quadratic, the global optimality of its solution can be guaranteed. Close proximity of the solution of the dynamic optimization problem with small activation and deactivation time constants and high tendon stiffness to the solution of the static optimization problem is therefore an indication of the optimality of the dynamic optimization solution. It is important to note here that optimality of the dynamic optimization solution of the problem with modified parameters does not guarantee optimality of the solution of the problem with original parameters. We resolved the dynamic optimization problem with activation and deactivation time constants of 5 ms instead of 15 and 60 ms, respectively, and by increasing the value of parameter *k*_*T*_ determining the steepness of the tendon force length characteristic from 35 to 1000 (see also online supplement). We then compared the solution of this limit problem to the solution of a corresponding static optimization problem. The static optimization problem was configured to match the dynamic optimization problem as closely as possible. The cost function was the integrand of the cost functional of the dynamic optimization problem evaluated at each time instant *i*, where muscle excitation was replaced by muscle activation. Muscle activations were bounded between 0 and 1 whereas the inputs for the ideal torque actuators—*e*_T*k*_—were bounded between -1 and 1. Pre-computed muscle moment arms were used to relate the tendon forces and ideal torques to the inverse dynamics joint reaction torques (Eq. 11). Muscle activation and tendon force were linearly related through Eqs. (4) and (7) using a rigid tendon with length $l_{\text{T}}^{s}$. The static optimization problem was solved using MATLAB’s lsqlin.

## Results

**Figure 4:**
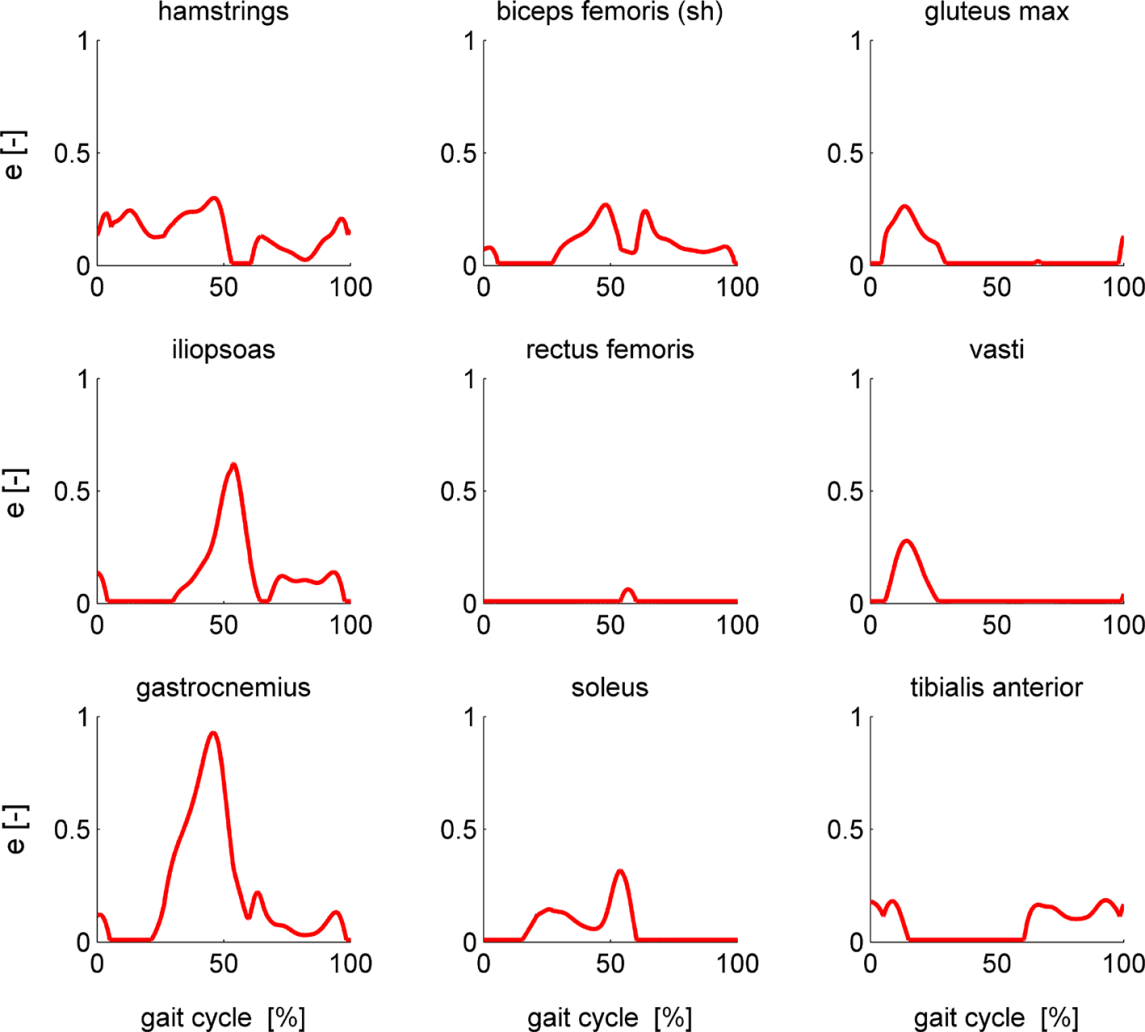
Optimal muscle excitations for the nine muscles of the simple model computed using formulation 1 (black), formulation 2 (orange), formulation 3 (gray), and formulation 4 (red). The different solutions nearly coincide.

**Figure 5:**
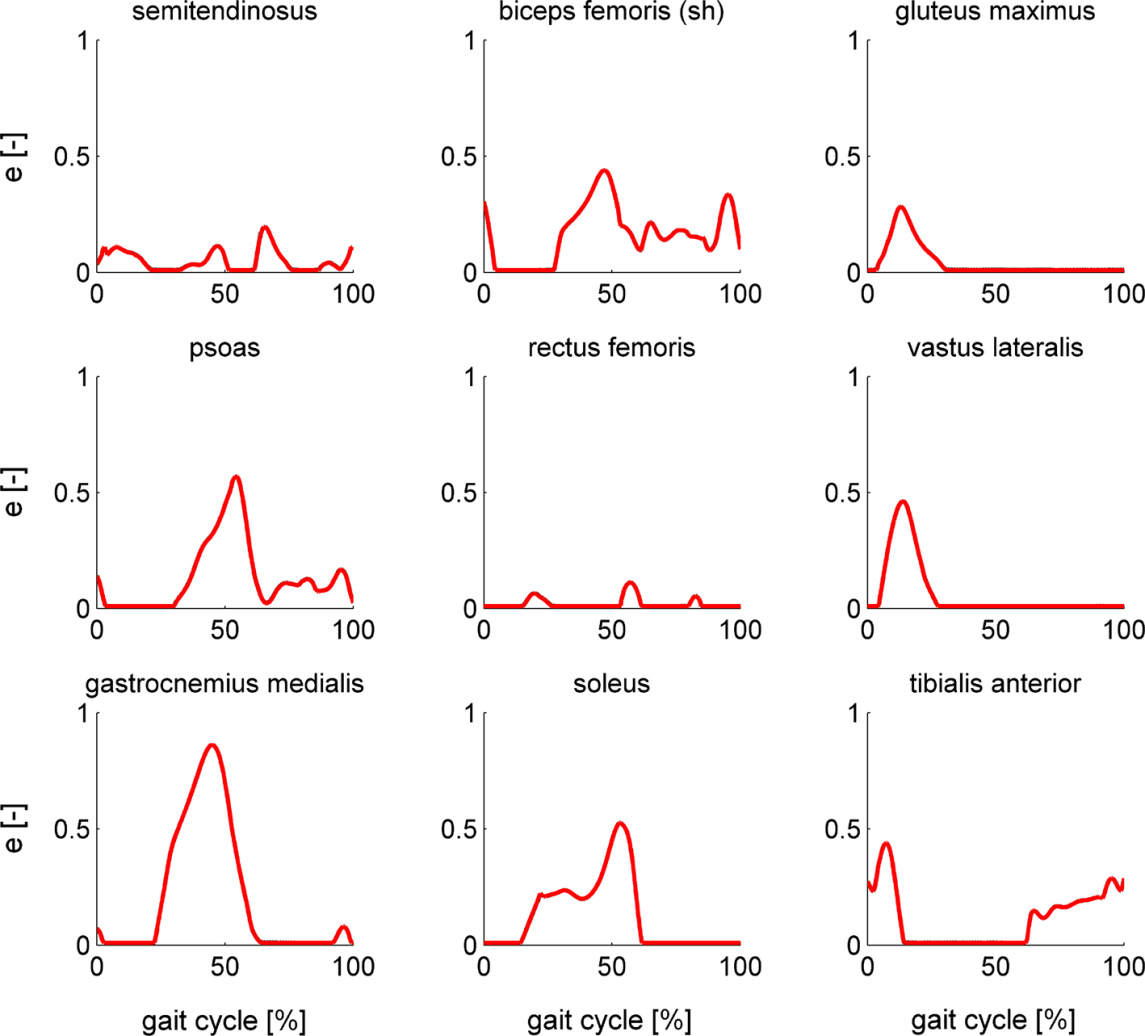
Optimal muscle excitations for a subset of the muscles of the complex model computed using formulation 1 (black), formulation 3 (gray), and formulation 4 (red). The different solutions nearly coincide.

Optimal control problem formulation influenced convergence (Tables 1, 2). Only formulations 3 and 4, which used extra controls and an implicit formulation of contraction dynamics, converged for all conditions evaluated in this study. Convergence of formulation 2, which used normalized fiber length as a state, was poorest. The different formulations converged to nearly identical optimal muscle excitations for both the simple (Fig. 4) and complex model (Fig. 5). In all cases, only the lower bounds on muscle excitations and muscle activations were active. The contributions of the ideal torque actuators to the inverse dynamics torques were always smaller than 0.7 Nm. These ideal torques do not exceed what is expected given measurement and modeling uncertainty. The reader is referred to the online supplement for figures of ideal torques and muscle activations and tendon forces of all muscles of the complex model.

**Table 1:** Comparison of different problem formulations for the simple model.

Formulation	Hot start				Arbitrary initial guess			
	1	2*	3	4	1	2	3	4
Convergence	Yes	Yes	Yes	Yes	Yes	No	Yes	Yes
Optimal value	0.3339	0.3378	0.3336	0.3385	0.3339	–	0.3339	0.3385
Accuracy	0.0024	0.0044	0.0024	0.0026	0.0024	–	0.0024	0.0026
CPU time (s)	15	193	13	22	27	–	7	45
Robustness	3.59*e*−6	–	1.17*e*−4	1.75*e*−7	3.59*e*−6	–	1.17*e*−4	1.75*e*−7

*Formulation 2 did not converge from the hot start but converged from the optimal solution of formulation 4

**Table 2:** Comparison of different problem formulations for the complex model.

Formulation	Hot start				Arbitrary initial guess			
	1	2	3	4	1	2	3	4
Convergence	Yes	No	Yes	Yes	No	No	Yes	Yes
Optimal value	0.9600	–	0.9590	0.957	–	–	0.9591	0.9589
Accuracy	0.0021	–	0.020	0.0020	–	–	0.0020	0.0020
CPU time (s)	1727	–	1937	1389	–	–	988	2723
Robustness	–	–	8.90*e*−5	1.89*e*−4	–	–	8.90*e*−5	1.89*e*−4

Robustness could not be computed for formulation 1, since convergence was not obtained for the arbitrary initial guess

For the simple model, all formulations except formulation 2 converged from both the hot start and the arbitrary initial guess. Formulation 2 converged when given the optimal solution of formulation 4 as an initial guess. Optimal solutions of the different formulations and for different initial guesses were nearly identical as can be seen from the cost function values and robustness against initial guess in Table 1 and from the optimal muscle excitation patterns in Fig. 4. Mesh accuracy was similar for formulations 1, 3, and 4. CPU times were between 7 and 45 s with formulation 3 having the lowest CPU times.

For the complex model, only formulations 3 and 4 converged from both the hot start and the arbitrary initial guess. Formulation 1 converged only from the hot start and formulation 2 did not converge from either the hot start or the arbitrary initial guess (Table 2). Optimal solutions of the different formulations and for different initial guesses were nearly identical, as can be seen from the cost function values and robustness against initial guess in Table 2 and from the optimal muscle excitation patterns in Fig. 5. Mesh accuracy was similar for formulations 1, 3, and 4. CPU times were between 988 and 2723 s with formulation 3 having the lowest CPU times.

Allowing activations to drop to zero for formulations 3 and 4, which did not require dividing by muscle activation to evaluate contraction dynamics, had a small but positive effect on mesh accuracy and almost always reduced computation time (Tables 3, 4).

**Table 3:** Comparison of formulations 3 and 4 with muscle activation bound between 0 and 1 for the simple model.

Formulation	Hot start		Arbitrary initial guess	
	3	4	3	4
Convergence	Yes	Yes	Yes	Yes
Optimal value	0.2970	0.3013	0.2969	0.3013
Accuracy	0.0023	0.0024	0.0023	0.0024
CPU time (s)	11	11	6	36
Robustness	1.15*e*−4	1.61*e*−7	1.15*e*−4	1.61*e*−7

**Table 4:** Comparison of formulations 3 and 4 with muscle activation bound between 0 and 1 for the complex model.

Formulation	Hot start		Arbitrary initial guess	
	3	4	3	4
Convergence	Yes	Yes	Yes	Yes
Optimal value	0.8647	0.8643	0.8646	0.8643
Accuracy	0.0019	0.0019	0.0019	0.0019
CPU time (s)	1520	1037	1170	2025
Robustness	9.46*e*−5	8.91*e*−6	9.46*e*−5	8.91*e*−6

Both optimality tests confirmed the close proximity of the numerical solutions to the optimal solution of the dynamic optimization problems. First, the costate approximations at the initial time matched the finite difference approximation of the sensitivity of the cost with respect to the initial state (Table 5). Second, the RMS difference between the optimal activations obtained by static and dynamic optimization decreased by a factor ten from 0.0223 to 0.0027 when activation and deactivation time constants were decreased and tendon stiffness was increased (formulation 3, activations bounded between 0 and 1, 100 mesh intervals), showing that the dynamic optimization solution approximated the static optimization solution when the two approaches were made similar. With a further decrease in time constants and increase in tendon stiffness, no accurate solution was obtained on a mesh with 100 intervals due to the increased stiffness of muscle activation and contraction dynamics.

**Table 5:** Post-optimality results for formulations 3 and 4.

	HAM	BFsh	GM	IP	RF	VA	GA	SOL	TA
Formulation 3—simple model									
$\lambda_{a}^{*} (t_{0} )$	−0.0059	−0.0014	0.0021	−0.0027	0.0026	0.0069	−0.0030	0.0049	−0.0052
Δ*J*/Δ*a*	−0.0058	−0.0013	0.0021	−0.0027	0.0030	0.0075	−0.0030	0.0048	−0.0051
Formulation 4—simple model									
$\lambda_{a}^{*} (t_{0} )$	−0.0059	−0.0014	0.0021	−0.0027	0.0026	0.0069	−0.0030	0.0049	−0.0052
Δ*J*/Δ*a*	−0.0058	−0.0015	0.0022*	−0.0025	0.0031	0.0076	−0.0030	0.0050	−0.0051

Analysis was performed for formulations 3 and 4 with muscle activation bound between 0 and 1 for the simple model. $\lambda_{a}^{*} (t_{0} )$ is the optimal costate of muscle activation at the initial time, and Δ*J*/Δ*a* is the ratio of the change in cost to the change in initial activation. Muscle names are abbreviated: HAM for hamstrings, BFsh for biceps femoris short head, GM for gluteus maximus, IP for iliopsoas, VA for vasti, GA for gastrocnemii, SOL for soleus, and TA for tibialis anterior

* Computed with a Δ*a* of 0.0005 instead of 0.001

## Discussion

This study evaluated four possible optimal control problem formulations for solving the muscle redundancy problem while taking muscle activation and contraction dynamics into account. Although all formulations converged from at least one initial guess for the simple model, the formulations that used explicit contraction dynamics failed to converge for all cases that were evaluated in this study (Tables 1, 2). The formulation with explicit contraction dynamics and normalized fiber length as the state variable was especially sensitive to the initial guess. In contrast, the two formulations that used implicit contraction dynamics converged to an optimal solution in all cases for all initial guesses. These findings suggest that use of implicit contraction dynamics may result in the most robust formulation of the dynamic optimization problem when using direct collocation.

Introducing additional controls that are proportional to the time derivative of the states resulted in very simple dynamic equations. The nonlinear equations describing contraction dynamics were then imposed as algebraic path constraints in their implicit form. By using the implicit form of the Hill model, evaluation of contraction dynamics did not require inversion of normalized force-velocity curves. In combination with the bounds on the controls and states, this formulation always resulted in well-bounded values for all variables in the Hill model (Eqs. 3–7), which may have helped convergence. This well-bounded nature could not be guaranteed for formulations that used explicit contraction dynamics and required inversion of the force-velocity characteristic in combination with unbounded values for the state (tendon force or muscle fiber length) derivatives. Furthermore, formulations that used tendon force as a state generally converged faster due to fewer NLP iterations. This result might be explained by the more linear relationship between muscle activation and tendon force than between muscle activation and muscle fiber length.

An additional advantage of these implicit formulations is that muscle activations are allowed to drop to zero, since no division by muscle activation is required to evaluate contraction dynamics. When muscle activation is small, the equilibrium between muscle force and tendon force (substitute Eqs. 3–4 into Eq. 7) defines muscle fiber velocity poorly since the fiber velocity dependent term is multiplied by a small value for activation. When muscle activation is zero, however, muscle length is fully determined by the equilibrium between passive muscle force and tendon force. This observation may explain why imposing lower bounds of 0 instead of 0.01 on muscle activations had a positive effect on computation times.

Although direct collocation methods for solving the muscle redundancy problem have been explored in previous studies,^1^,^2^,^7^,^8^,^29^ this study is the first to investigate the influence of different problem formulations on the accuracy and robustness of the numerical solution. The results of this study are especially important since optimality of the obtained solutions was never checked previously confirming the statement of Hicks *et al.* that verification of numerical methods used to solve for the unknowns in a simulation is often overlooked.^12^ In addition, robustness as well as numerical challenges related to the convergence of gradient-based solvers have been identified as an important limitation to the use of dynamic optimization by non-experts.^14^,^29^ The close proximity of the numerical solutions to the optimal solution of the dynamic optimization problems was confirmed two ways. In addition, the direct collocation solutions of the formulations that used implicit contraction dynamics have low dependence on the initial guess and hence these formulations can be considered to be robust. Moreover, the low dependence on the initial guess is an indication that there is no need to use global optimization methods for these particular problems. Results from two really different initial guesses were reported—a hot start that can be obtained in a few CPU seconds^8^ and arbitrary constant values in time for all controls and states. We did not explore the use of a completely random initial guess, since better than random initial guesses are readily available.

Direct collocation is a computationally efficient alternative to direct shooting methods, which are commonly used. In contrast to shooting methods that typically require large computation times and often do not converge to an optimal solution, we obtained convergence in 7 to 45 s of CPU time for a simple model and 16–45 min of CPU time for a complex model. Using automatic differentiation reduced CPU times by about a factor of ten compared to using finite difference derivatives. Automatic differentiation is an alternative for numerical or symbolic differentiation. Numerical differentiation by finite differences requires multiple function evaluations and is less accurate due to the finite approximation. Symbolic differentiation also results in analytic derivatives but has the disadvantage of being sensitive to the complexity of the function.^23^ The increase in computational efficiency when using automatic differentiation followed from the reduced CPU time in NLP function evaluations and for the formulations with fiber length as a state also from the reduced number of NLP iterations. The reduced number of NLP iterations might be explained by the higher gain in accuracy for the formulations with fiber length as a state that rely on the highly non-linear relation between muscle activity and fiber length. Additional advantages of our method over direct shooting methods are that we do not need to use a simple parametrization of the controls (e.g., block patterns^20^) to keep the problem tractable, and we can easily assess the accuracy of the numerical solution.

Direct comparison of CPU times with results from the literature is difficult, since in contrast to the majority of reported studies, we used an inverse dynamics instead of forward dynamics approach for skeletal dynamics. Since skeletal dynamics was solved by an inverse dynamics analysis preceding our optimizations, only muscle dynamics instead of muscle plus skeletal dynamics was evaluated during the optimization. As a result, computational efficiency was increased. In addition, CPU times are influenced by problem formulation and the specific motion being tracked. Nevertheless, Menegaldo *et al.* 15 needed about 55 min of CPU time to solve a similar dynamic optimization problem preceded by an inverse dynamics analysis of skeletal motion for a simple three degree-of-freedom planar model with ten muscles based on direct shooting, whereas we required less than a minute of CPU time for a model of comparable complexity.

Van den Bogert *et al.* 29 have previously used direct collocation in combination with an implicit formulation of muscle contraction dynamics, and although their findings about computational efficiency were similar to ours, they found that convergence depended critically on the availability of a good initial guess. There are several possible reasons for this difference in robustness. First, van den Bogert *et al.* use a forward instead of inverse dynamics approach to solve for skeletal motion, resulting in a harder dynamic optimization problem. Second, we not only used an implicit formulation of contraction dynamics but also introduced additional controls defined as the state derivatives, which were bounded. The well-bounded nature of the optimization problem might have improved the robustness to the initial guess. Third, a more accurate collocation method—Legendre-Guass-Radau quadrature instead of midpoint Euler—was used in this study.

The proposed muscle dynamic optimization approach is a robust alternative for computed muscle control (CMC), a popular approach to compute dynamically consistent muscle controls that track a given motion.^28^ Instead of solving a dynamic optimization problem, CMC uses static optimization along with feedforward and feedback control to drive a musculoskeletal model towards the experimentally measured kinematics. However, due to the combination of static optimization and a forward simulation of muscle and skeleton dynamics based on time-marching, muscle forces computed with CMC are extremely sensitive to model parameter values (e.g., segment mass and inertia) and the instant in time at which the simulation is started.^32^ In contrast, our approach is robust against small changes in model parameter values because of the low sensitivity of inverse dynamics simulations to mass and inertia parameters^32^ and the absence of time-marching. In addition, the muscle force solution does not depend on the initial and final time except for a short time interval of about 50 ms at the beginning and end of the motion due to the unknown initial and final state. The computational efficiency of the direct collocation method proposed in this paper is comparable to CMC and hence the use of a robust, dynamic optimization method instead of CMC comes at no additional cost.

**Figure 6:**
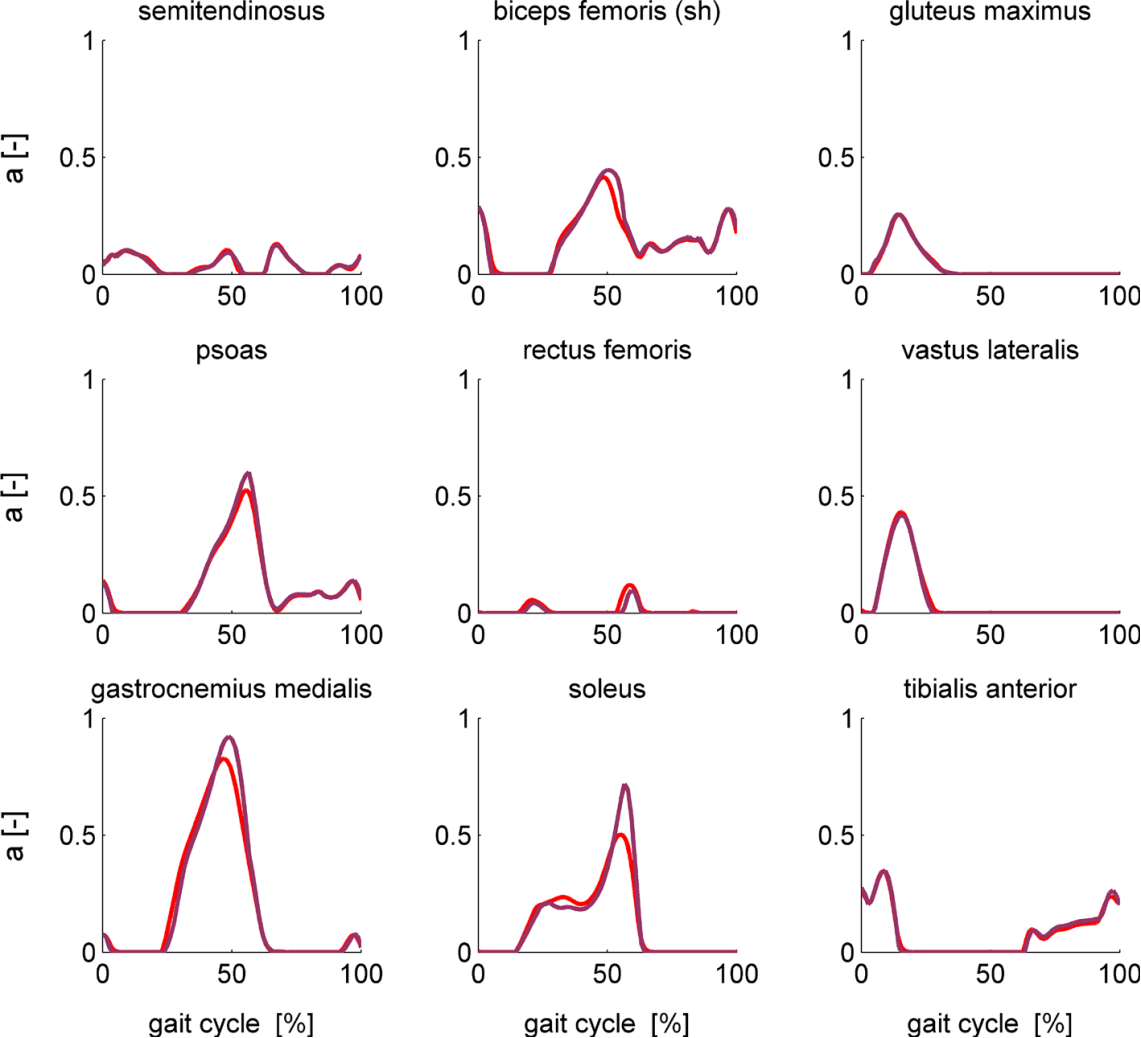
Comparison of muscle activations during walking computed based on static optimization (purple) and formulation 4 of the muscle dynamic optimization problem (red) for a subset of the muscles of the complex model.

**Figure 7:**
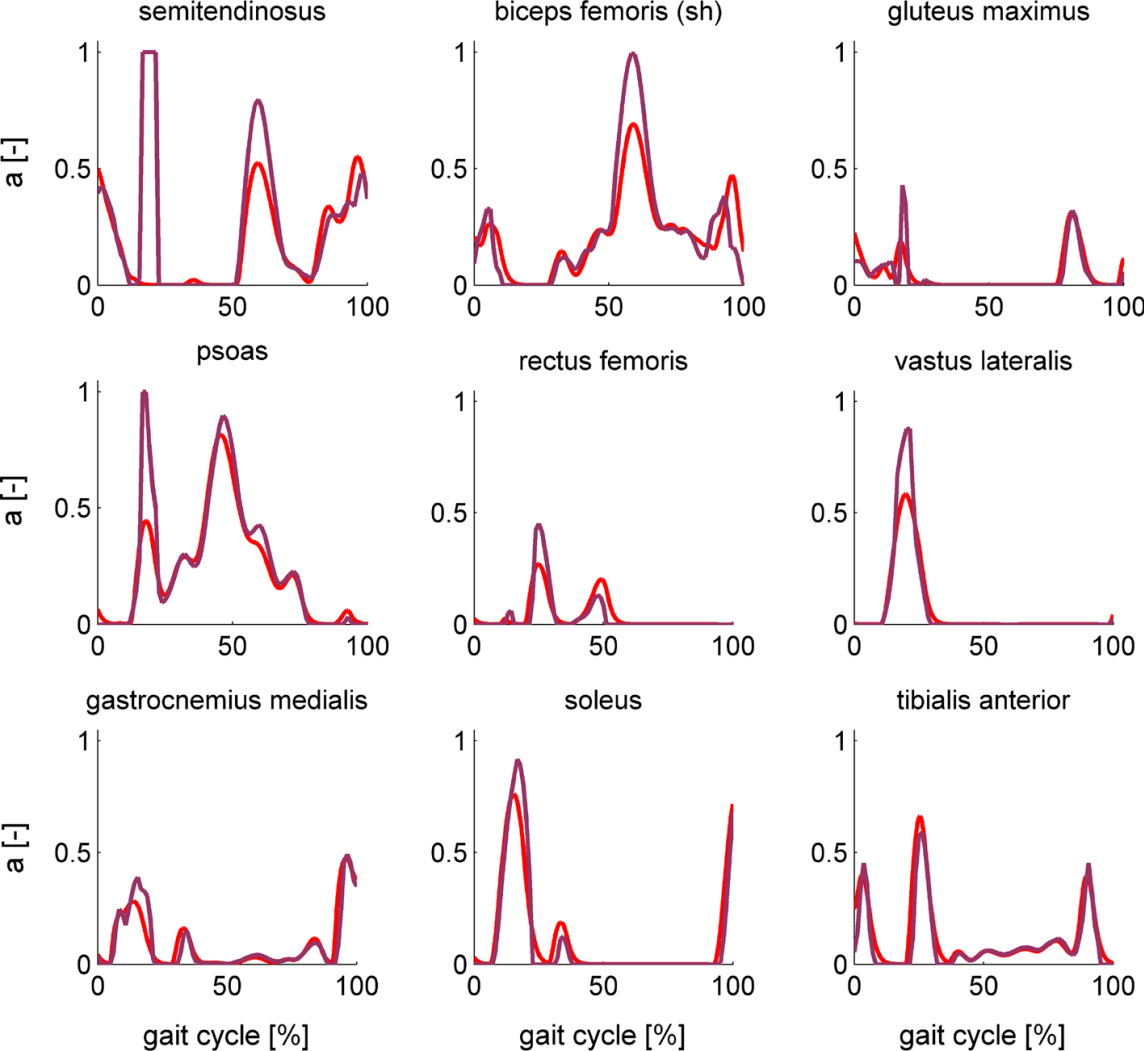
Comparison of muscle activations during running computed based on static optimization (purple) and formulation 4 of the muscle dynamic optimization problem (red) for a subset of the muscles of the complex model.

Computation times are still considerably higher for dynamic than for static optimization and hence modeling of muscle dynamics should be motivated by the research question. Some have argued that static and dynamic optimization yield similar muscle forces during walking^5^ and even running.^14^ To illustrate that this similarity should be assessed in light of the research question, we compared static and dynamic optimization solutions for walking and running. Solutions for running were obtained by applying the same models and methods on one cycle of treadmill running data collected at 9.5 km/h from a male test subject (64.8 kg, 1.76 m). The subject provided written informed consent in accordance with the ethical committee of UZ Leuven. Static and dynamic optimization yielded different muscle activations during walking for muscles with long, compliant tendons such as the gastrocnemii and soleus, where the rigid tendon assumption of static optimization is less valid (Fig. 6). The corresponding muscle forces, however, were very similar for the two optimization approaches. Hence, a compliant tendon allows generating the same amount of force with lower muscle activation by allowing the muscle to operate closer to its optimal fiber length and hence augments the efficiency of the muscle. These results suggest that modeling of muscle dynamics may be important to study muscle efficiency during walking but may not have a large influence on the computation of joint contact forces, which are mainly determined by muscle forces. Modeling muscle dynamics is more important to study faster motions such as running where the neglect of muscle dynamics limits the performance of the model, resulting in maximal muscle activity for some muscles (Fig. 7) and different muscle force predictions for static and dynamic optimization. This finding is in accordance with Miller *et al.*,^16^ who found that sprinting performance significantly decreases in the absence of tendon compliance. Hence, we conclude that modeling muscle dynamics may be important to assess efficiency and performance, even in slow motions, and to assess muscle forces in faster motions. In addition, the use of muscle dynamic optimization instead of static optimization enables using time-dependent cost functions such as metabolic energy consumption; studying the effect of tendon stiffness, which is especially relevant in elderly and athletes; including history dependent muscle dynamics; and accounting for muscle state feedback.

An important limitation of our study is that we used inverse dynamics to solve skeletal dynamics, which is applicable only if skeletal motion is known *a priori*. Previously, forward dynamic approaches have been used to assess muscle contributions to a measured motion (e.g.,^20^). This approach was possibly motivated by the lack of inverse dynamic methods that accounted for muscle dynamics at that time. Our method provides a computationally efficient alternative for forward dynamic approaches that track a known motion, but it cannot replace forward dynamic approaches that are used to predict optimal motion patterns. Note that under the assumption of zero tracking errors and equal contribution of the ideal torque actuators, the use of an inverse and forward dynamic analysis of skeletal motion to estimate muscle forces for a given motion are equivalent, i.e., they result in the same muscle excitation patterns. Our inverse dynamic approach, however, does not allow non-zero tracking errors. We plan to extend the proposed direct collocation method to include skeletal dynamics, enabling predictive simulations in the future. Another limitation of this study is that results for only two motion cycles were reported. However, the method worked equally well when applied to additional data (walking, running, and perturbed standing of different subjects).

The use of gradient-based optimization methods requires that objective and constraint functions are twice continuously differentiable. Therefore we had to use a smooth approximation of the activation dynamics model proposed by Winters *et al.* 33 We investigated the influence of the parameter b defining the smoothness of the transition between activation and deactivation dynamics on the optimal solution and found that it was small. Similarly, all normalized muscle force-length and force-velocity characteristics need to be smooth and twice continuously differentiable. Characteristics proposed in the literature vary widely. In our problems, use of a steeper normalized muscle force-velocity curve and of smaller activation and deactivation time constants required a finer mesh to obtain the same accuracy and resulted in higher computation times.

In conclusion, we evaluated different optimal control problem formulations for computing dynamically consistent muscle controls that reproduce inverse dynamics joint torques during walking. Optimal control problem formulation mainly influenced convergence and CPU time. The formulations that used implicit muscle dynamics in combination with additional controls allowed for a robust solution of the muscle redundancy problem for a 3D musculoskeletal model with 43 muscles per leg in about 20 min of CPU time. The close proximity of the numerical solutions to the optimal solution of the dynamic optimization problem was confirmed in two ways. Our approach, which is based on direct collocation, is orders of magnitude faster than direct shooting approaches that have been used previously to compute muscle inputs that track a measured motion. Hence, direct collocation in combination with the proposed implicit formulation of contraction dynamics is a computationally efficient and robust alternative to direct shooting methods for solving dynamic optimization problems with motion tracking. Therefore, this approach might enable the use of dynamic optimization by non-experts seeking to investigate the effect of muscle dynamics on efficiency and optimal performance. Future work should focus on comparing the present approach to other approaches for computing muscle forces. The present approach lacks some of the major limitations of established methods such as static optimization and CMC while remaining computationally efficient.

## Electronic Supplementary Material

Supplementary Material (PDF 2323 KB)
