# Supplementary material for: Evaluation of Direct Collocation Optimal Control Problem Formulations for Solving the Muscle Redundancy Problem
Source: Ann Biomed Eng. 2016 Mar 21;44(10):2922–36. doi: 10.1007/s10439-016-1591-9 (PMC5043004; doi:10.1007/s10439-016-1591-9)
Supplement: Supplementary file 1 — Supplementary Material (PDF 2323 KB) [file 10439_2016_1591_MOESM1_ESM.pdf]

EVALUATION OF DIRECT COLLOCATION OPTIMAL CONTROL PROBLEM FORMULATIONS FOR SOLVING THE MUSCLE REDUNDANCY PROBLEM
Online Supplement

Friedl De Groote, Allison L. Kinney, Anil V. Rao, Benjamin J. Fregly

1 Mathematical expressions for muscle-tendon characteristics

Properties of muscle and tendon were described by dimensionless characteristics. All characteristics are at least second order continuous and the tendon force-length characteristic f_t is third order continuous for compatibility with the interior point numerical optimization solver that uses second order derivative information. Third order continuity of f_t is required, since the first derivative of the tendon-force length curve is used in formulations 1 and 3 (see below). f_t is described by an exponential function:

$$f_t(\tilde{l}_T) = c_1 \exp[k_T(\tilde{l}_T - c_2)] - c_3, \tag{S1}$$

with $\tilde{l}_T$ normalized tendon length, $k_T = 35$ tendon stiffness at 4% strain, and coefficients c_1, c_2, and c_3. The active force-length characteristic f_{act} is described by the sum of three Gaussian functions:

$$f_{act}(\tilde{l}_M) = \sum_{i=1}^{3} b_{1i} \exp\left[\frac{-0.5(\tilde{l}_M - b_{2i})^2}{b_{3i} + b_{4i}\tilde{l}_M}\right], \tag{S2}$$

with $\tilde{l}_M$ normalized fiber length and coefficients b_{1i}, b_{2i}, b_{3i}, and b_{4i} for $i = 1 \ldots 3$. The passive force-length characteristic f_{pas} is described as in OpenSim's Thelen2003Muscle by an exponential function:

$$f_{pas}(\tilde{l}_M) = \frac{\exp\left(\frac{k_{\text{pe}}(\tilde{l}_M - 1)}{e_0}\right) - 1}{\exp(k_{\text{pe}}) - 1}. \tag{S3}$$

The force-velocity characteristic f_v is described by a logarithmic function:

$$f_v(\tilde{v}_M) = d_1 \log[(d_2\tilde{v}_M + d_3) + \sqrt{((d_2\tilde{v}_M + d_3)^2 + 1)}] + d_4, \tag{S4}$$

with $\tilde{v}_M$ normalized fiber velocity and coefficients d_1, d_2, d_3, and d_4. Numerical values for all coefficients are reported in Table 1.

Tendon force-length	k_T	35
	c_1	0.200
	c_2	0.995
	c_3	0.250
Active muscle force-length	b_{11}	0.815
	b_{21}	1.055
	b_{31}	0.162
	b_{41}	0.063
	b_{12}	0.433
	b_{22}	0.717
	b_{32}	-0.030
	b_{42}	0.200
	b_{13}	0.100
	b_{23}	1.000
	b_{33}	0.354
	b_{43}	0.000
Passive muscle force-length	k_{pe}	4.0
	e_0	0.6
Muscle force-velocity	d_1	-0.318
	d_2	-8.149
	d_3	-0.374
	d_4	0.886

Table 1: Parameters of the Hill model characteristics.

2 Full-form expressions of constraints imposing muscle dynamics

Contraction dynamics was imposed using four different formulations. To this aim, four different functions were derived from the Hill-model described by equations 3-7 (see manuscript). Full-form expressions for these functions are given below.

Formulation 1

In formulation 1, contraction dynamics is imposed using normalized tendon force $\tilde{F}_T = \frac{F_T}{F_M^0}$ as a state:

$$\frac{\mathrm{d}\tilde{F}_T}{\mathrm{d}t} = f_1(a, \tilde{F}_T) \tag{S5}$$

f_1 can be found by taking the first time derivative of eq. 3:

$$\frac{\mathrm{d}\tilde{F}_T}{\mathrm{d}t} = \frac{\mathrm{d}\tilde{f}_t}{\mathrm{d}t}(\tilde{l}_T) = c_1 k_T \mathrm{exp}\left[k_T(\tilde{l}_T - c_2)\right]\frac{\mathrm{d}\tilde{l}_T}{\mathrm{d}t}. \tag{S6}$$

Evaluating eq. S6 requires normalized tendon length $\tilde{l}_T$ and normalized tendon velocity $\tilde{v}_T = \frac{\mathrm{d}\tilde{l}_T}{\mathrm{d}t}$. $\tilde{l}_T$ can be solved from eq. 3 using $\tilde{F}_T$, which is an input to function f_1:

$$\tilde{l}_T = f_t^{-1}(\tilde{F}_T) = \frac{1}{k_T}\mathrm{log}\left[\frac{1}{c_1}(\tilde{F}_T + c_3)\right] + c_2. \tag{S7}$$

Tendon velocity v_T can be related to muscle-tendon velocity v_{MT} and fiber velocity v_M by differentiating eq. 5 with respect to time:

$$v_{MT} = v_T + v_M \mathrm{cos}\alpha - l_M \mathrm{sin}\alpha \frac{\mathrm{d}\alpha}{\mathrm{d}t}. \tag{S8}$$

Differentiating eq. 6 with respect to time yields:

$$v_M \mathrm{sin}\alpha + l_M \mathrm{cos}\alpha \frac{\mathrm{d}\alpha}{\mathrm{d}t} = 0, \tag{S9}$$

from which we can obtain an expression for $\frac{\mathrm{d}\alpha}{\mathrm{d}t}$:

$$\frac{\mathrm{d}\alpha}{\mathrm{d}t} = -\frac{v_M \mathrm{sin}\alpha}{l_M \mathrm{cos}\alpha}. \tag{S10}$$

Eq. S10 can then be substituted in eq. S8, which simplifies to the following expression:

$$v_{MT} = v_T + \frac{v_M}{\mathrm{cos}\alpha}. \tag{S11}$$

Hence, we find tendon velocity from:

$$v_T = v_{MT} - \frac{v_M}{\mathrm{cos}\alpha}, \tag{S12}$$

requiring fiber velocity v_M and the cosine of the pennation angle cosα. Normalized fiber velocity can be solved from eq. 4:

$$\tilde{v}_M = f_v^{-1}(\tilde{F}_M^v) = \frac{1}{d_2}\left(\sinh\left[\frac{1}{d_1}(\tilde{F}_M^v - d_4)\right]\right), \tag{S13}$$

with

$$\tilde{F}_M^v = \frac{\tilde{F}_M - f_{\text{pas}}(\tilde{l}_M)}{a f_{\text{act}}(\tilde{l}_M)}. \tag{S14}$$

l_M can be solved from eq. 5 and 6. These two equations can be rewritten as:

$$l_M \text{sin}\alpha = l_M^0 \text{sin}\alpha_0, \tag{S15}$$

$$l_M \text{cos}\alpha = l_{MT} - l_T. \tag{S16}$$

Squaring both sides and adding the resulting equations together eliminates α and results in the following equation for l_M:

$$l_M = \sqrt{(l_M^0 \text{sin}\alpha_0)^2 + (l_{MT} - l_T)^2}, \tag{S17}$$

which can be evaluated using l_T (eq. S7). The cosine of the pennation angle cosα can be solved from eq. 6:

$$\text{cos}\alpha = \frac{l_{MT} - l_T}{l_M}. \tag{S18}$$

F_M can be solved from eq. 7:

$$F_M = \frac{F_T}{\text{cos}\alpha} \tag{S19}$$

Summarized, f_1 is computed by subsequently evaluating eq. S7, denormalizing $\tilde{l}_T$ ($l_T = \tilde{l}_T l_T^S$), evaluating eq. S17, normalizing l_M ($\tilde{l}_M = \frac{l_M}{l_M^0}$), evaluating eqs. S18 and S19, normalizing F_M ($\tilde{F}_M = \frac{F_M}{F_M^0}$), evaluating eqs. S14 and S13, denormalizing $\tilde{v}_M$ ($v_M = \tilde{v}_M v_M^{max}$), evaluating eq. S12, normalizing v_T ($\tilde{v}_T = \frac{v_T}{l_T^S}$) and evaluating S6 using normalized tendon force $\tilde{F}_T$ and muscle activation a as inputs. Note that muscle-tendon length l_{MT} and muscle-tendon velocity v_{MT} are determined by the motion and are computed prior to solving the optimization problem. l_{MT} is computed using OpenSim's Muscle Analysis and v_{MT} is obtained by differentiating the cubic spline interpolation of l_{MT} in MATLAB.

Formulation 2

In formulation 2, contraction dynamics is imposed using normalized fiber length $\tilde{l}_M$ as a state:

$$\frac{\text{d}\tilde{l}_M}{\text{d}t} = f_2(a, \tilde{l}_M) \tag{S20}$$

f_2 can be found by taking the time derivative of normalized fiber length:

$$\frac{\text{d}\tilde{l}_M}{\text{d}t} = \frac{1}{l_M^0}\frac{\text{d}l_M}{\text{d}t} = \frac{1}{l_M^0} v_M = \frac{v_M^{\text{max}}}{l_M^0}\tilde{v}_M \tag{S21}$$

Normalized fiber velocity can be solved from eq. 4 (see eqs. S13 and S14). Normalized fiber length $\tilde{l}_M$ is an input to f_2. Computing muscle force F_M from eq. S19 requires F_T and $\cos\alpha$. To compute tendon force F_T from eq. 2, tendon length l_T is required. Tendon length l_T can be solved from eqs. 5 and 6. Squaring both sides of eq. 6 and substituting $1-\cos^2\alpha$ for $\sin^2\alpha$ yields:

$$l_M^2(1-\cos^2\alpha) = (l_M^0 \sin\alpha_0)^2. \tag{S22}$$

Solving this equation for $l_M\cos\alpha$ and substituting the result in eq. 5 yields:

$$l_T = l_{MT} - \sqrt{\left[l_M^2 - (l_M^0 \sin\alpha_0)^2\right]}. \tag{S23}$$

$\cos\alpha$ is then obtained from eq. S18.

Summarized, f_2 is computed by subsequently denormalizing $\tilde{l}_M$, evaluating eq. S23, normalizing l_T, evaluating eqs. S18, 3 and S19, normalizing F_M, evaluating eqs. S14, S13 and S21.

Formulation 3

In formulation 3, contraction dynamics is imposed using normalized tendon force $\tilde{F}_T$ as a state and introducing u_F, the scaled time derivative of the normalized tendon force, as a new control simplifying the contraction dynamic equations:

$$\frac{\mathrm{d}\tilde{F}_T}{\mathrm{d}t} = s_F u_F. \tag{S24}$$

The Hill model was then imposed as a path constraint:

$$f_3(a, \tilde{F}_T, u_F) = 0. \tag{S25}$$

f_4 is obtained by substituting eq. 4 in eq. 7:

$$F_M^0\left[a f_{\text{act}}(\tilde{l}_M) f_v(\tilde{v}_M) - f_{\text{pas}}(\tilde{l}_M)\right]\cos\alpha - F_M^0 \tilde{F}_T = 0. \tag{S26}$$

Evaluating eq. S26 requires $\tilde{l}_M$, $\cos\alpha$ and $\tilde{v}_M$ in addition to inputs a and $\tilde{F}_T$. Fiber length $\tilde{l}_M$ can be computed by subsequently evaluating eq. S7, denormalizing $\tilde{l}_T$ and evaluating eq. S17. The cosine of the pennation angle $\cos\alpha$ can then be computed from eq. S18. Normalized fiber velocity $\tilde{v}_M$ can be computed from the relation between tendon, fiber, and muscle-tendon velocity (eq. S11):

$$v_M = (v_{MT} - v_T)\cos\alpha. \tag{S27}$$

To obtain tendon velocity v_T, eq. S6 is solved for $\tilde{v}_T = \frac{\mathrm{d}\tilde{l}_T}{\mathrm{d}t}$:

$$\tilde{v}_T = \frac{\frac{\mathrm{d}\tilde{F}_T}{\mathrm{d}t}}{c_1 k_T \exp\left[k_T(\tilde{l}_T - c_2)\right]}. \tag{S28}$$

Evaluating this equation requires $\frac{\mathrm{d}\tilde{F}_T}{\mathrm{d}t}$ and $\tilde{l}_T$. $\frac{\mathrm{d}\tilde{F}_T}{\mathrm{d}t}$ can be solved from eq. S24 and input u_F and $\tilde{l}_T$ was computed in the process of obtaining $\tilde{l}_M$.

Summarized, f_3 is computed by subsequently evaluating eq. S7, denormalizing $\tilde{l}_T$, evaluating eq. S17, normalizing l_M, evaluating eqs. S18 and S28, denormalizing $\tilde{v}_T$, evaluating eq. S27, normalizing v_M, and evaluating eq. S26.

General settings	
derivatives.derivativelevel	second
ipoptoptions.tolerance	1e-6
ipoptoptions.maxiterations	2000
ipoptoptions.linear_solver	ma57
method	RPM-integration
**Mesh refinement**	
mesh.method	hp-PattersonRao
mesh.tolerance	1e-6
mesh.maxiteration	10
mesh.colpointsmin	3
mesh.colpointsmax	10

Table 2: GPOPS-II settings. Settings that are not specified here were kept at their default value. The mesh refinement settings only affect the post-optimality analysis, since all other problems were only solved on the initial mesh.

Formulation 4

In formulation 4, contraction dynamics is imposed using normalized muscle fiber length $\tilde{l}_M$ as a state and introducing u_v, the scaled time derivative of normalized muscle length, as a new control simplifying the contraction dynamic equations:

$$\frac{\mathrm{d}\tilde{l}_M}{\mathrm{d}t} = \frac{v_M^{\max}}{l_M^0} u_v. \tag{S29}$$

The Hill model was then imposed as a path constraint:

$$f_4(a, \tilde{l}_M, u_v) = 0. \tag{S30}$$

f_4 is obtained by substituting eqs. 3 and 4 in eq. 7:

$$F_M^0 \left[a f_{\mathrm{act}}(\tilde{l}_M) f_v(\tilde{v}_M) - f_{\mathrm{pas}}(\tilde{l}_M) \right] \cos\alpha - F_M^0 f_t(\tilde{l}_T) = 0. \tag{S31}$$

with $\tilde{v}_m = u_v$, a and $\tilde{l}_M$ inputs. $\tilde{l}_T = \frac{l_T}{l_T^S}$ can be computed from eq. S23 and $l_M = l_M^0 \tilde{l}_M$ and $\cos\alpha$ can subsequently be computed from eq. S18.

3 Algorithm settings

All non-default GPOPS-II settings are specified in Table 2.

4 Additional results

Computed muscle activations and tendon forces for all muscles of the complex model during walking and running are reported in Figures 1-12. Muscle activations and tendon forces

computed with static optimization and muscle dynamic optimization are shown. Only the results obtained with formulation 4 are reported, since all formulations gave very similar results when they converged to a locally optimal solution. Muscle activations computed using static and dynamic optimization during walking only differ for muscles with long tendons (Figures 1-3), whereas tendon forces computed using static and dynamic optimization during walking are very similar (Figures 4-6). However, both muscle activations and tendon forces computed using static and dynamic optimization during running differ considerably for some muscles (Figures 7-12). Note that we chose to report absolute muscle forces to represent the relative importance of the different muscles.

Ideal joint torques during walking and running for the complex model are reported in Figures 13 and 14. Ideal torques during walking are small, i.e. around zero for static optimization and below 0.7Nm for dynamic optimization. Ideal torques during running are larger, i.e. up to about 15Nm, and very similar for static and dynamic optimization.

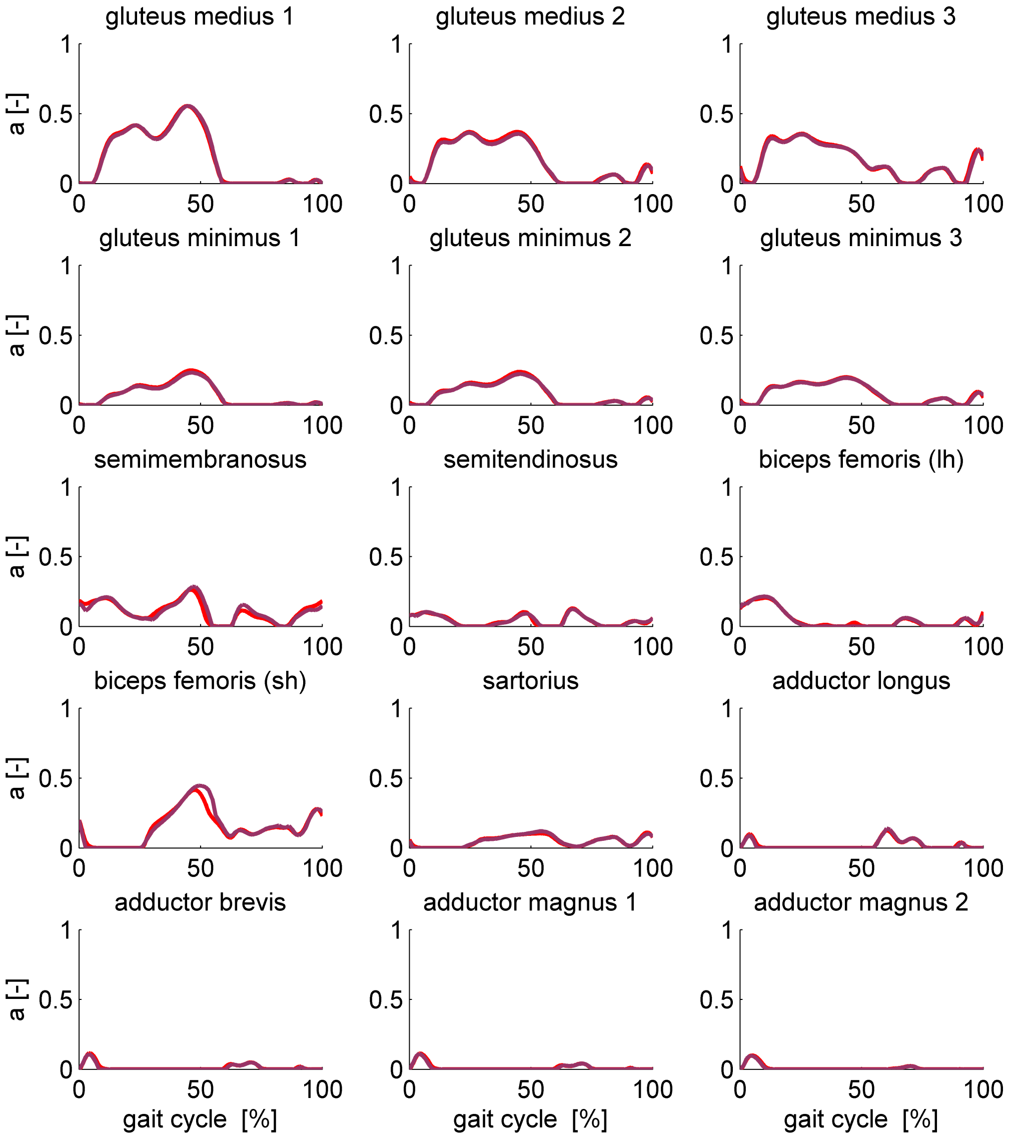

Figure 1: Comparison of muscle activations during walking computed based on static optimization (purple) and formulation 4 of the muscle dynamic optimization problem (red) - part 1.

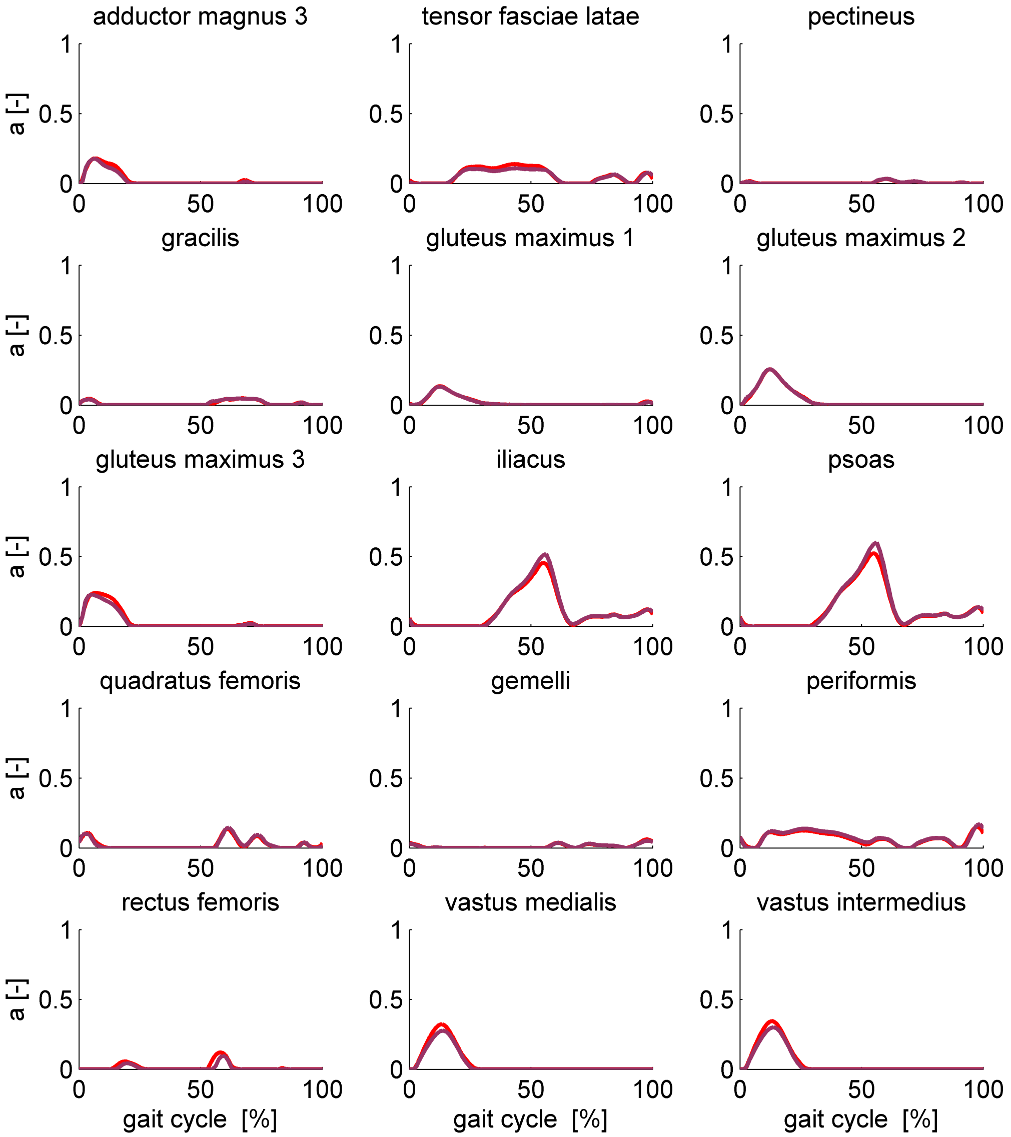

Figure 2: Comparison of muscle activations during walking computed based on static optimization (purple) and formulation 4 of the muscle dynamic optimization problem (red) - part 2.

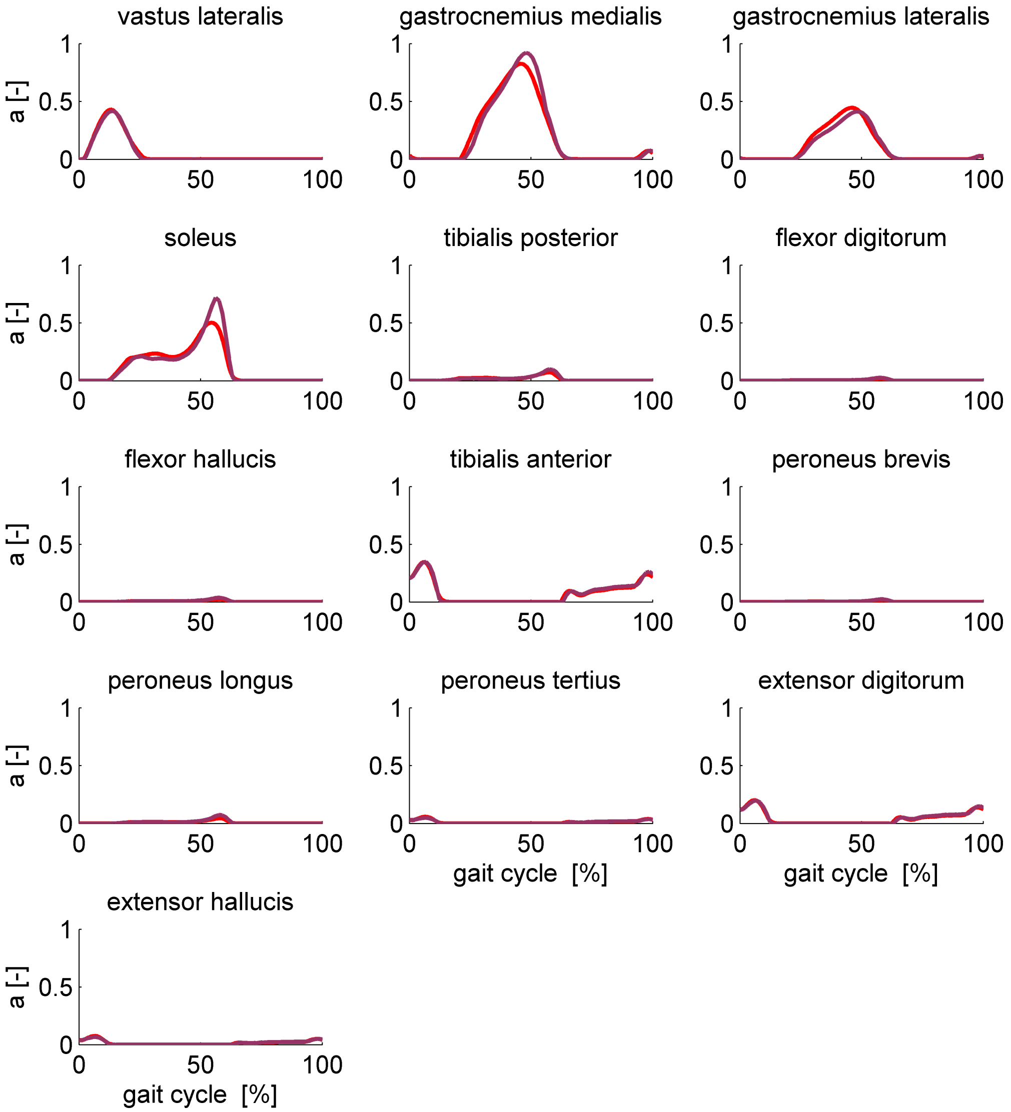

Figure 3: Comparison of muscle activations during walking computed based on static optimization (purple) and formulation 4 of the muscle dynamic optimization problem (red) - part 3.

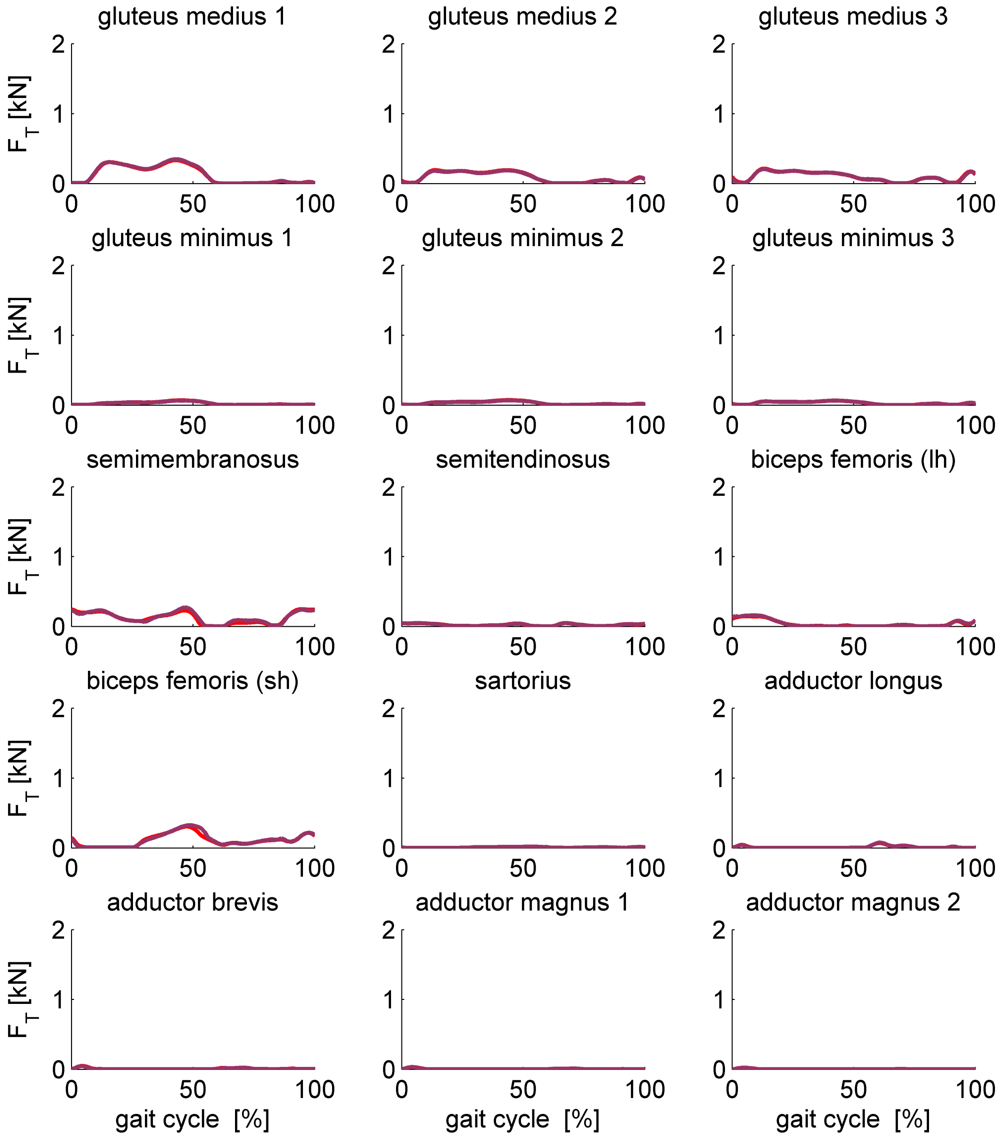

Figure 4: Comparison of tendon forces during walking computed based on static optimization (purple) and formulation 4 of the muscle dynamic optimization problem (red) - part 1. Note that units of force are kN.

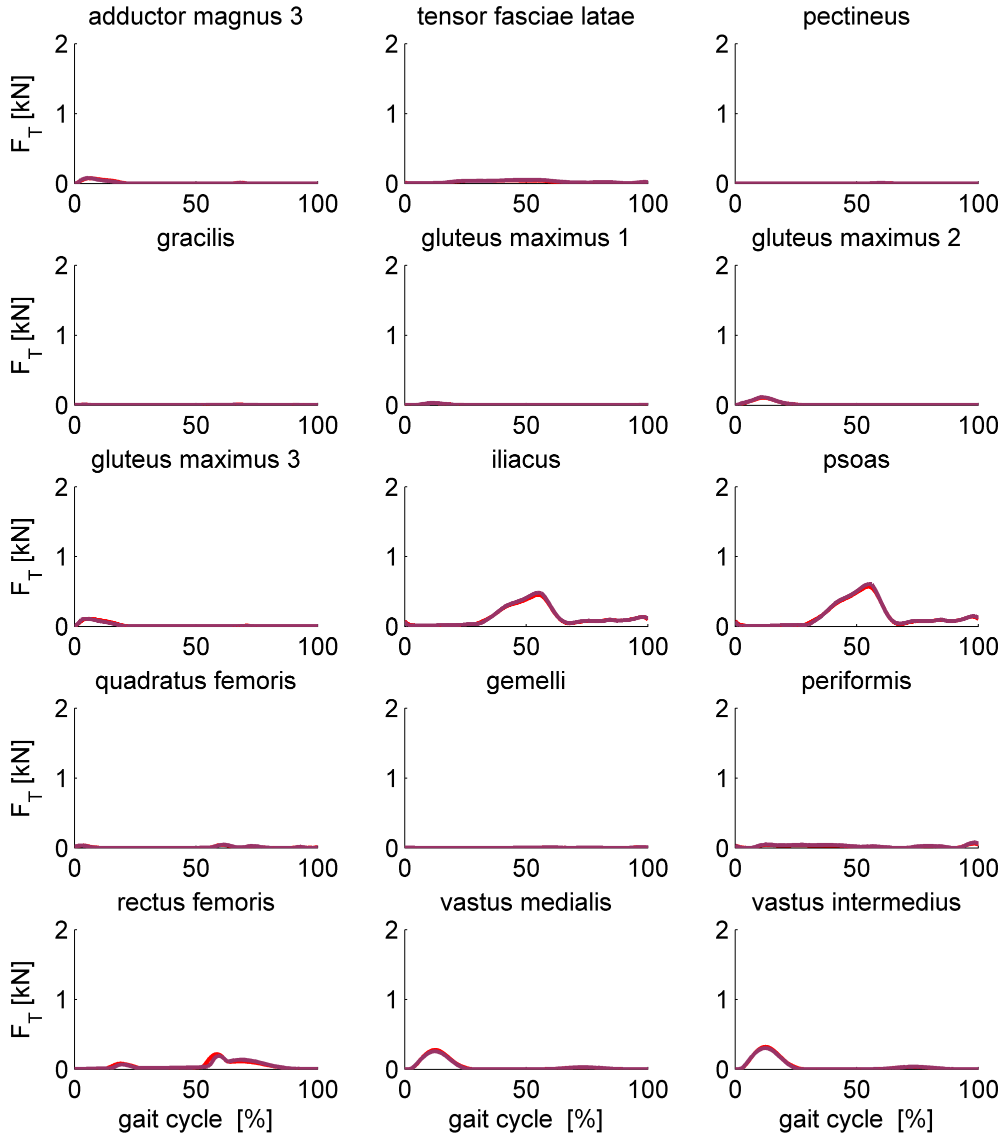

Figure 5: Comparison of tendon forces during walking computed based on static optimization (purple) and formulation 4 of the muscle dynamic optimization problem (red) - part 2. Note that units of force are kN.

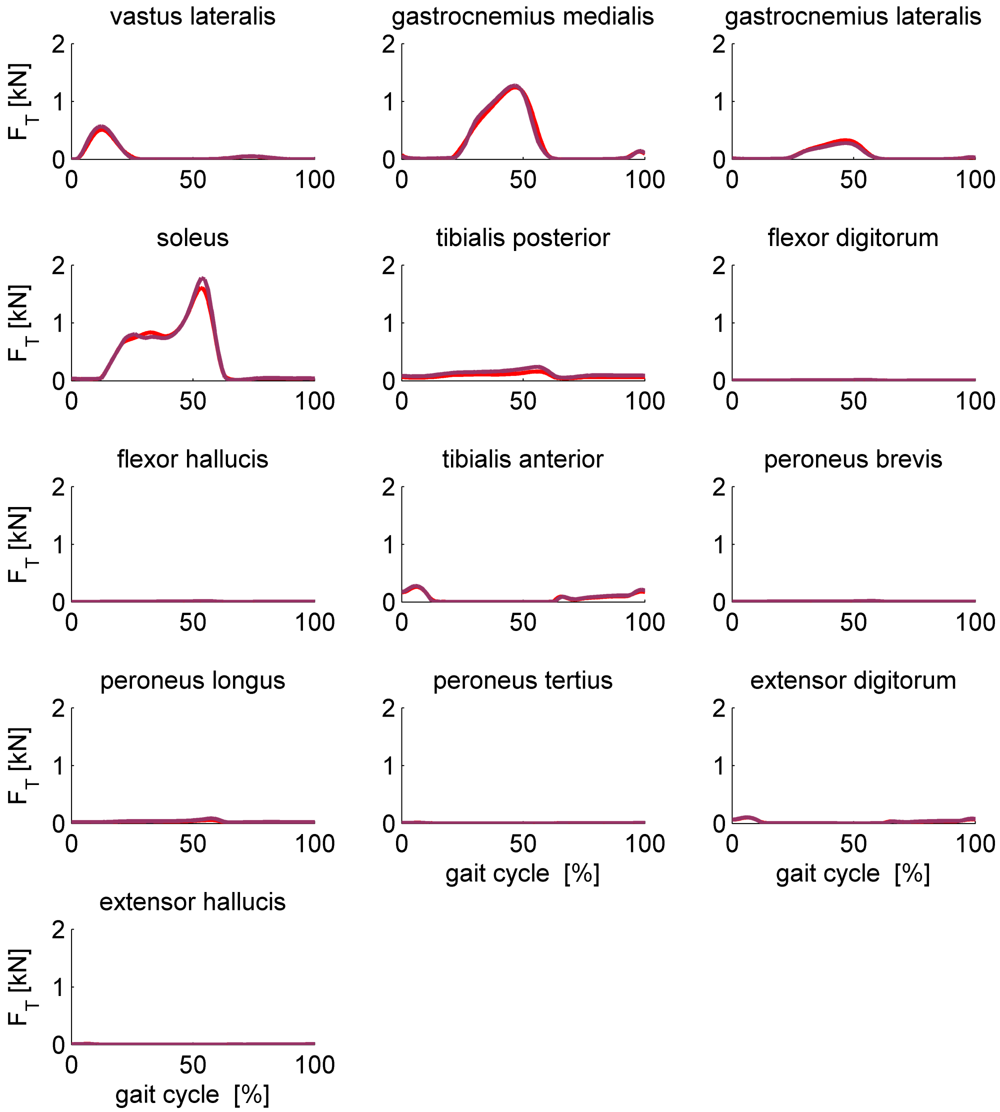

Figure 6: Comparison of tendon forces during walking computed based on static optimization (purple) and formulation 4 of the muscle dynamic optimization problem (red) - part 3. Note that units of force are kN.

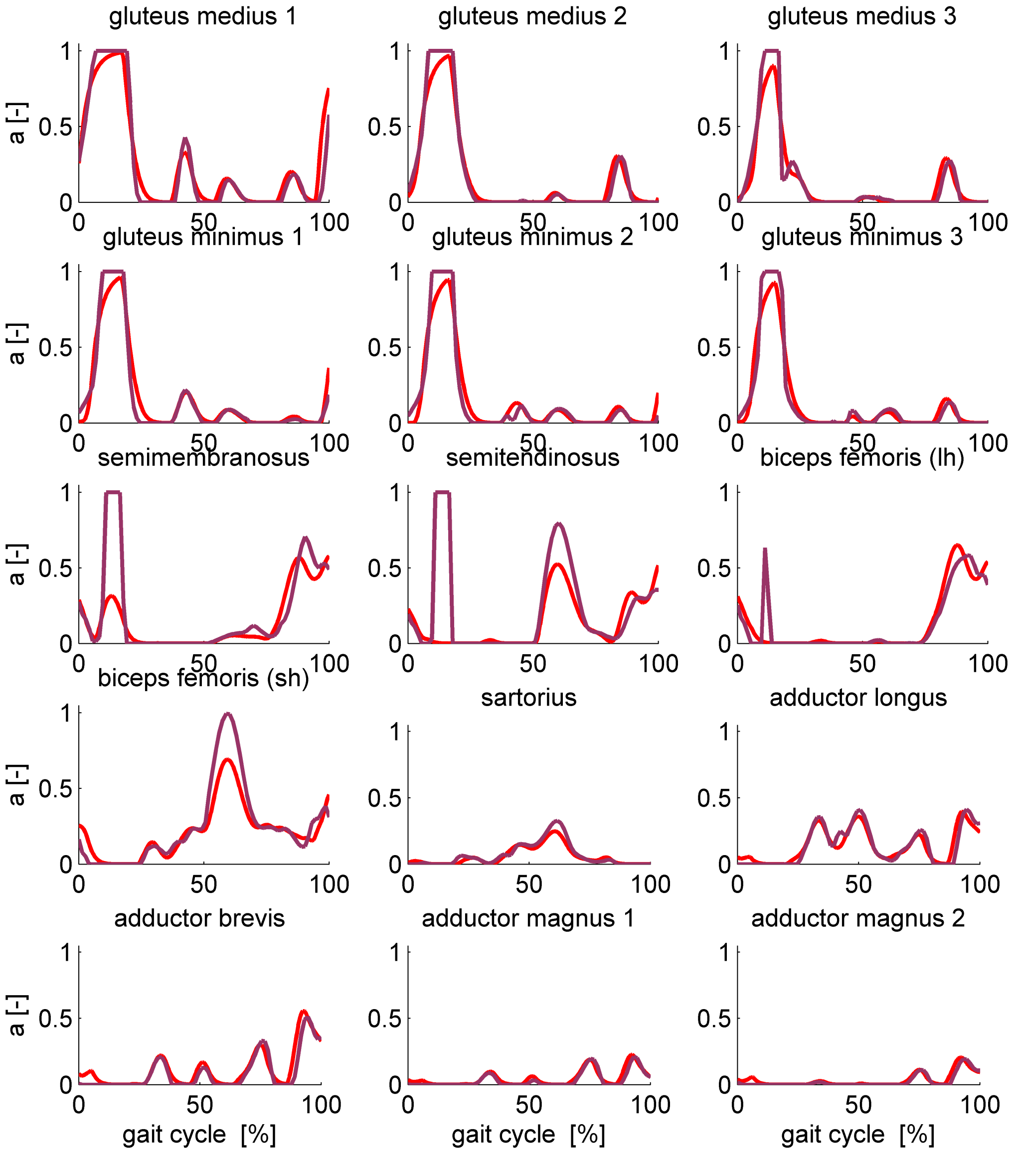

Figure 7: Comparison of muscle activations during running computed based on static optimization (purple) and formulation 4 of the muscle dynamic optimization problem (red) - part 1.

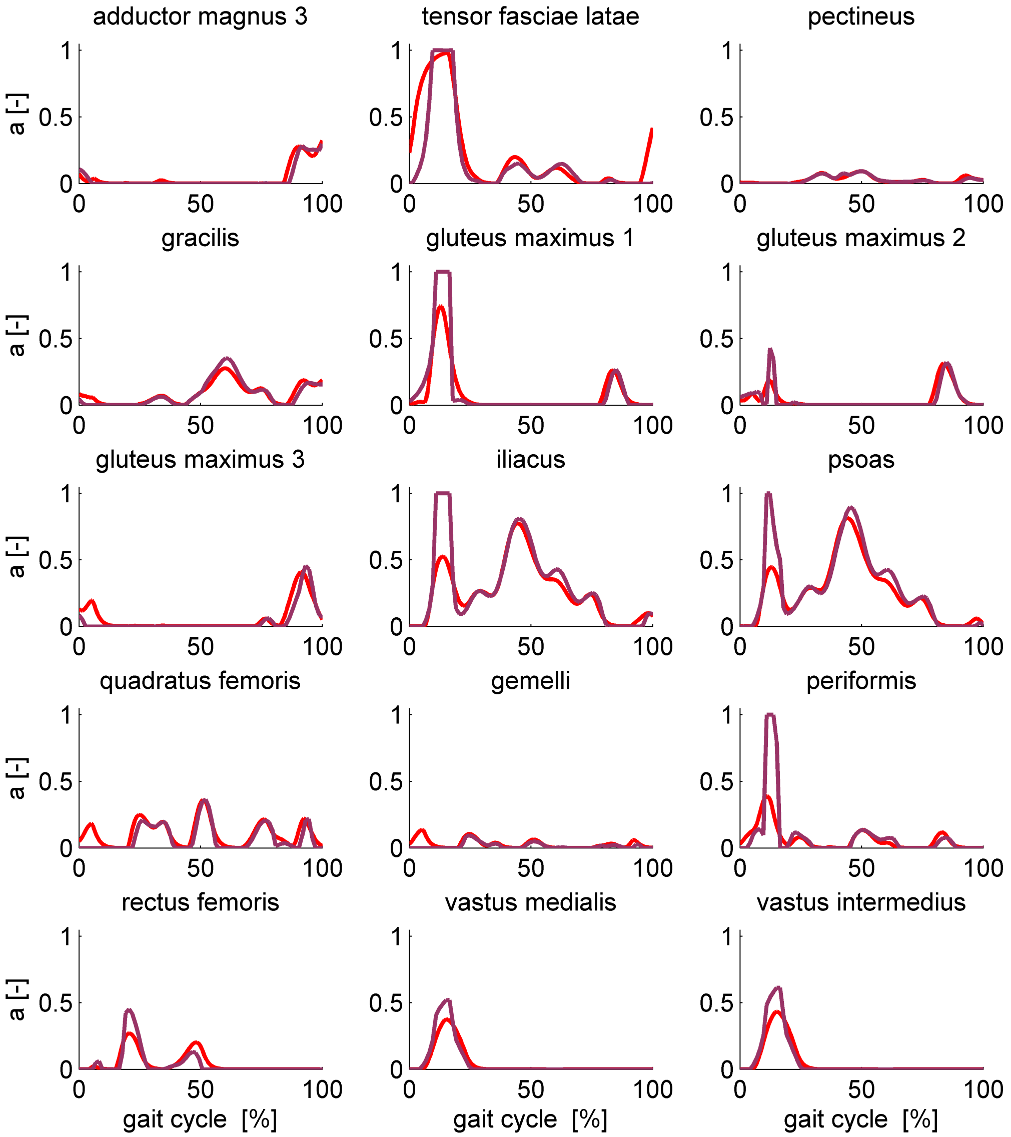

Figure 8: Comparison of muscle activations during running computed based on static optimization (purple) and formulation 4 of the muscle dynamic optimization problem (red) - part 2.

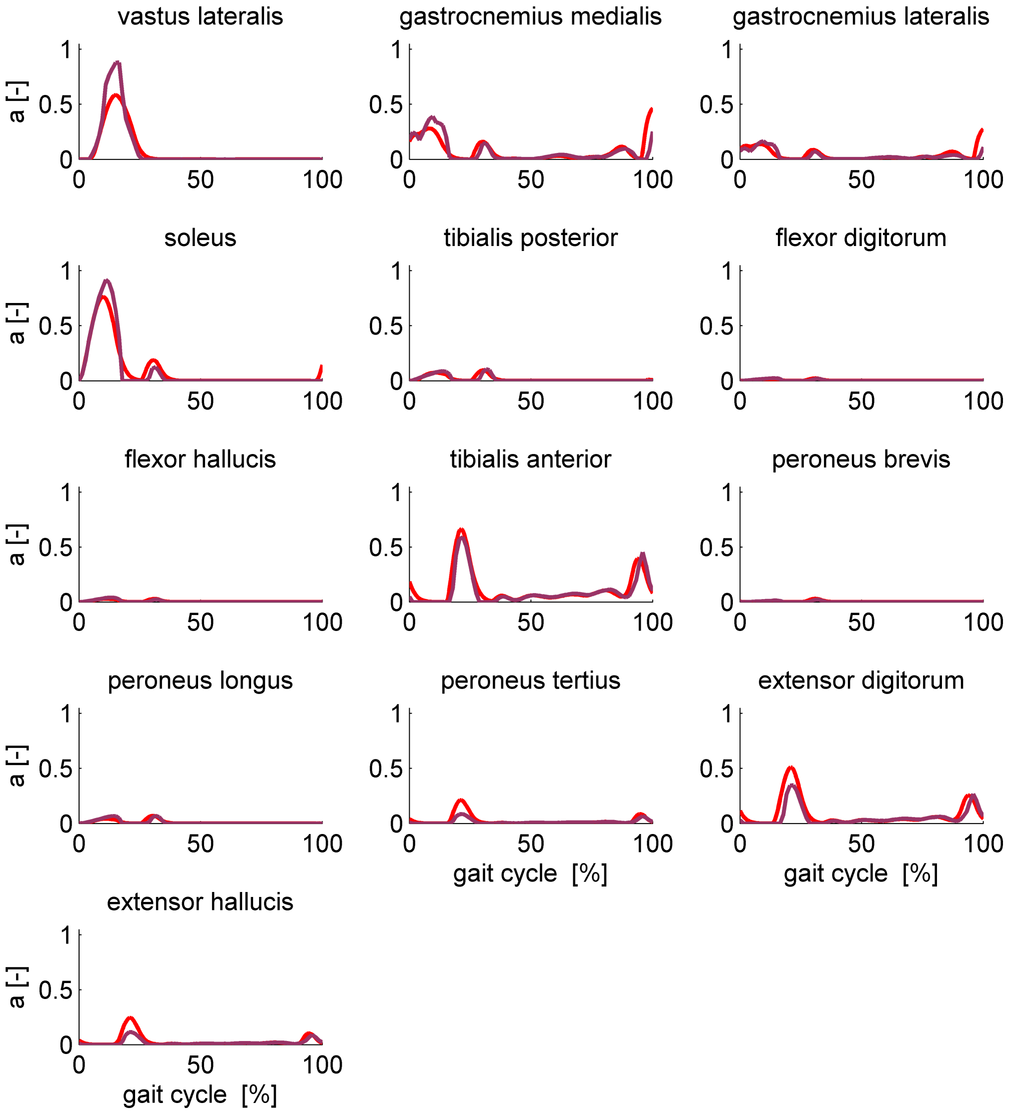

Figure 9: Comparison of muscle activations during running computed based on static optimization (purple) and formulation 4 of the muscle dynamic optimization problem (red) - part 3.

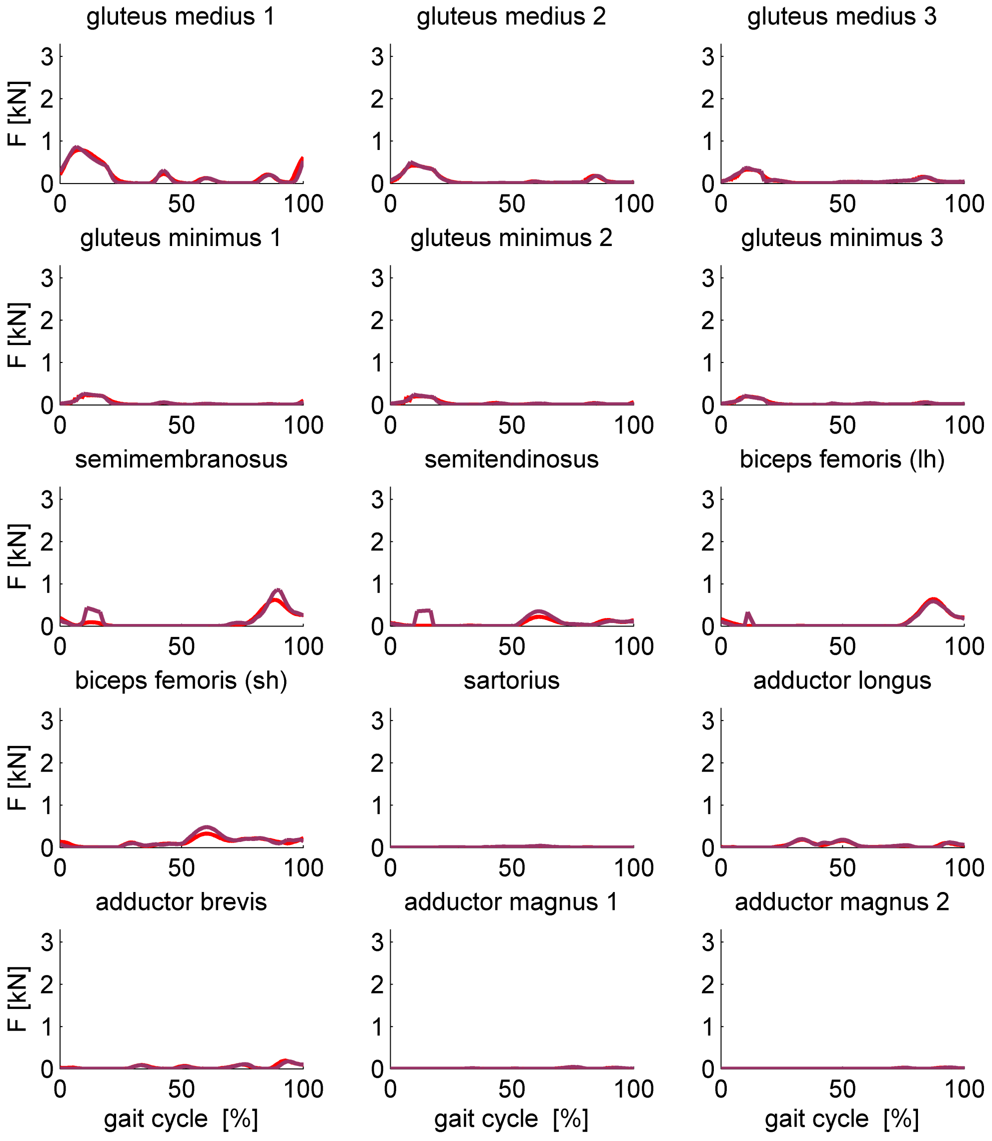

Figure 10: Comparison of tendon forces during running computed based on static optimization (purple) and formulation 4 of the muscle dynamic optimization problem (red) - part 1. Note that units of force are kN.

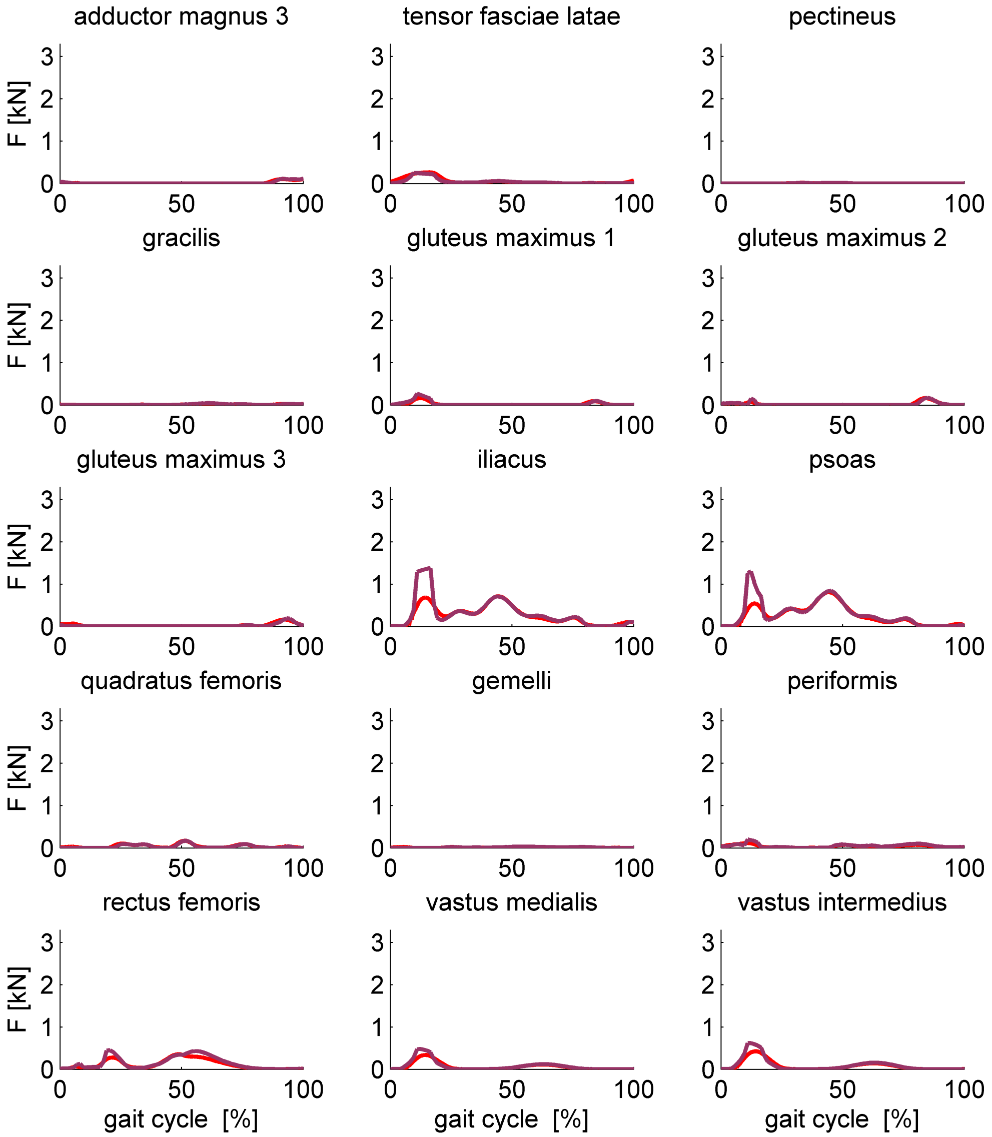

Figure 11: Comparison of tendon forces during running computed based on static optimization (purple) and formulation 4 of the muscle dynamic optimization problem (red) - part 2. Note that units of force are kN.

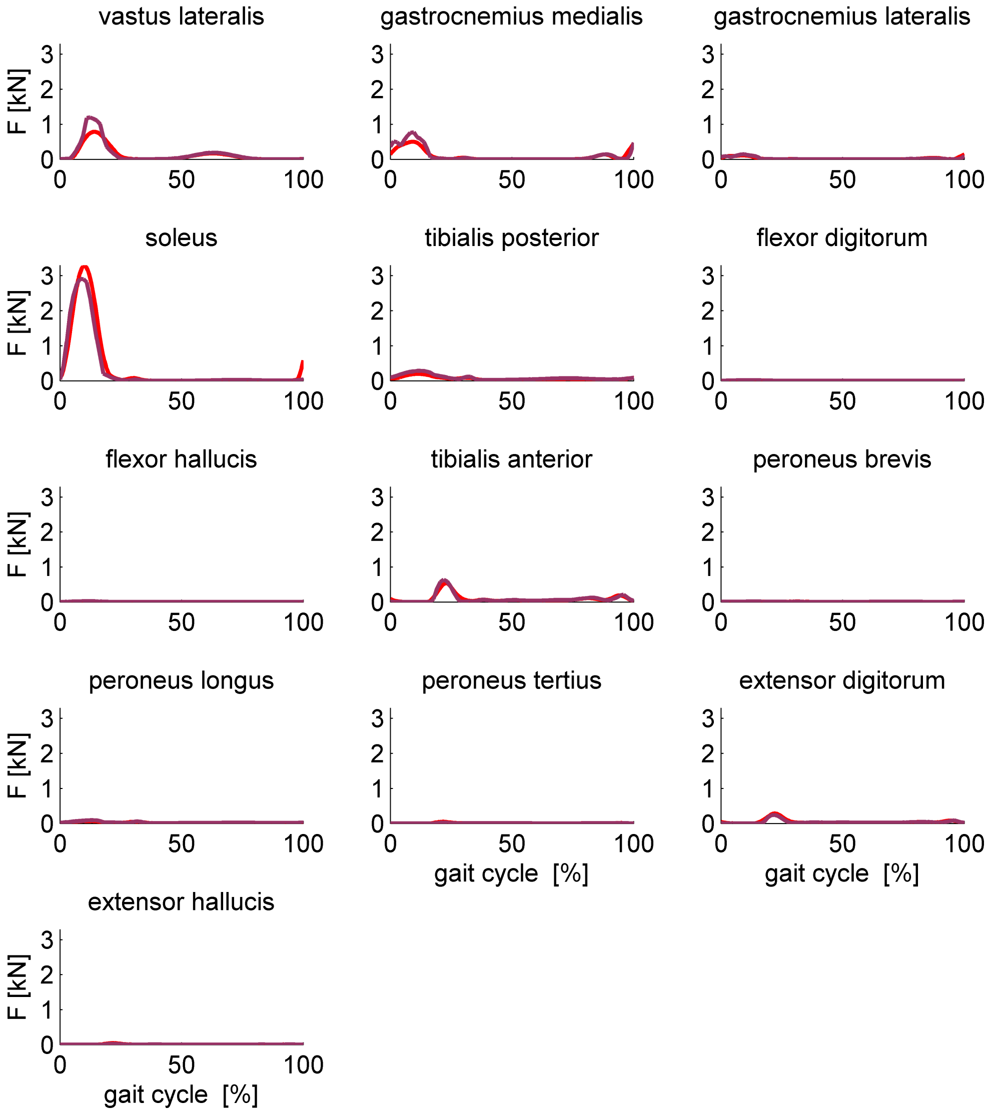

Figure 12: Comparison of tendon forces during running computed based on static optimization (purple) and formulation 4 of the muscle dynamic optimization problem (red) - part 3. Note that units of force are kN.

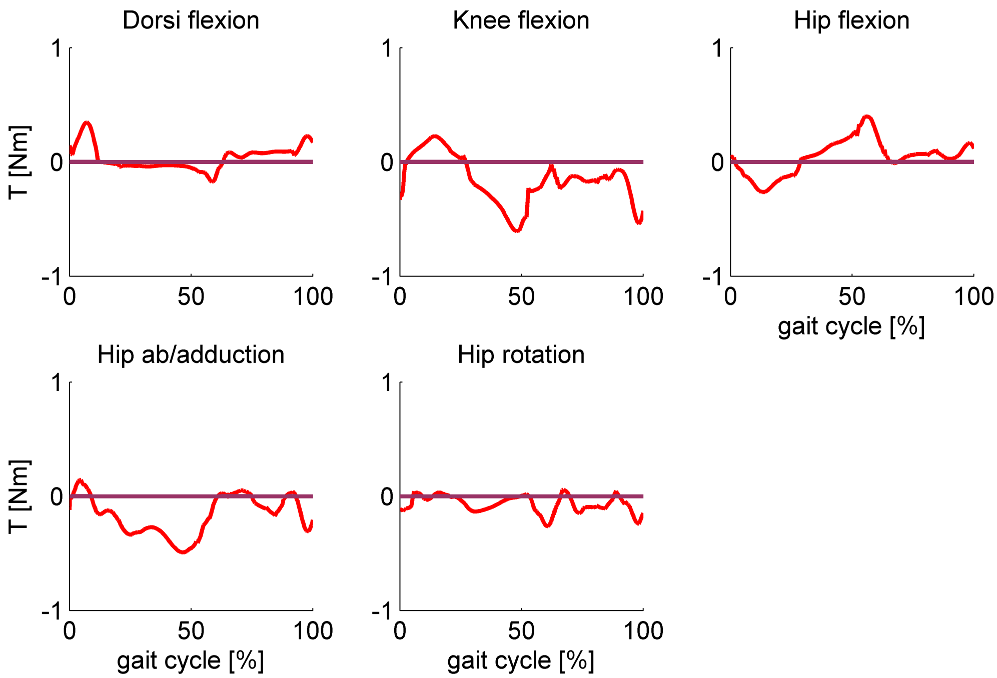

Figure 13: Ideal torques during walking computed using static optimization (purple) and formulation 4 of the muscle dynamic optimization problem (red). Internal ankle dorsiflexion, knee extension, hip flexion, hip adduction, and hip endorotation are positive.

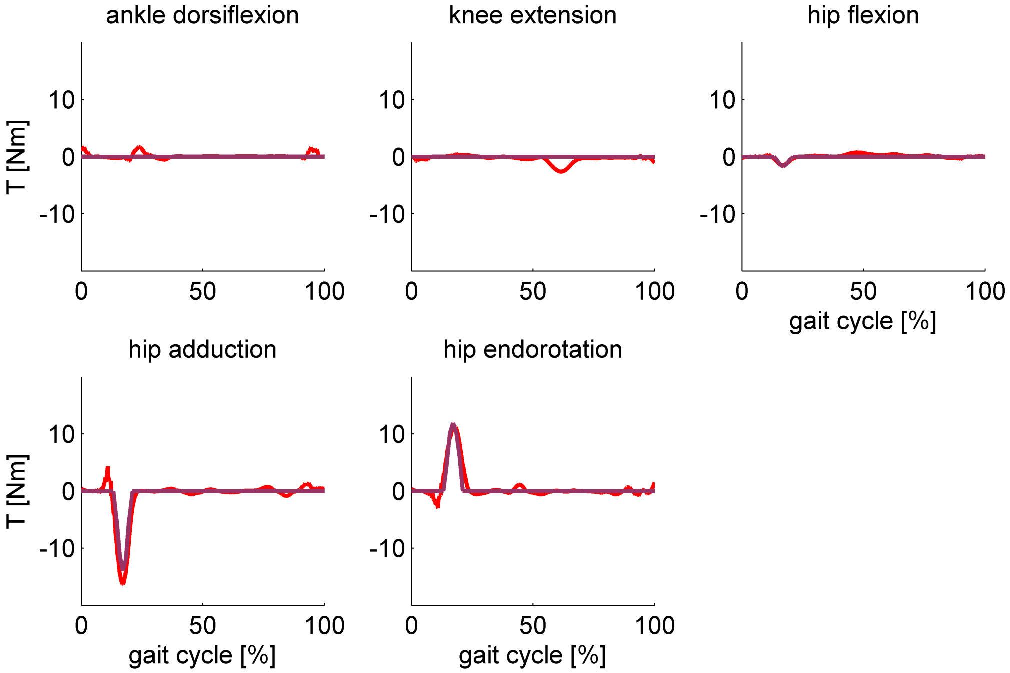

Figure 14: Ideal torques during running computed using static optimization (purple) and formulation 4 of the muscle dynamic optimization problem (red). Internal ankle dorsiflexion, knee extension, hip flexion, hip adduction, and hip endorotation are positive.
